# Sustainable Phosphorylated Cellulose Nanocrystals: A Dual‐Affinity Platform for High‐Efficiency Enrichment of Intact Glycopeptides and Phosphopeptides

**DOI:** 10.1002/advs.202523858

**Published:** 2026-02-27

**Authors:** Jiaying Li, Wuming Fan, Xuyang Yue, Dongdong Wang, Xiangyu Tang, Zhuo Zhang, Yonggui Wang, Yanjun Xie, Mingliang Ye, Hongqiang Qin

**Affiliations:** ^1^ College of Materials Science and Engineering Key Laboratory of Bio‐based Material Science and Technology (Ministry of Education) Northeast Forestry University Harbin China; ^2^ State Key Laboratory of Fine Chemicals School of Chemical Engineering Dalian University of Technology Dalian China; ^3^ CAS Key Laboratory of Separation Science For Analytical Chemistry Dalian Institute of Chemical Physics Chinese Academy of Sciences Dalian China

**Keywords:** dual‐affinity enrichment, glycoproteomics, phosphoproteomics, phosphorylated cellulose nanocrystals, post‐translational modifications (PTMs)

## Abstract

Protein glycosylation and phosphorylation are critical post‐translational modifications (PTMs) governing nearly all cellular functions, yet their analysis remains challenging due to reliance on costly and unsustainable enrichment materials. Herein, we report a green synthesis of phosphorylated cellulose nanocrystals (P‐CNCs) via one‐step phosphoric acid hydrolysis, enabling dual‐affinity enrichment of both glycopeptides and phosphopeptides. P‐CNCs leverage abundant surface hydroxyl groups for hydrophilic interaction liquid chromatography (HILIC)‐based glycopeptide capture, and intrinsic phosphate groups enable direct Ti^4^^+^ chelation (P‐CNCs‐Ti^4+^) for phosphopeptide enrichment without chemical derivatization. Using only 1 µL human serum, P‐CNCs captured 2,025 N‐linked and 2,183 O‐GalNAcylated glycopeptides, including 10 previously unreported N‐glycosylation chemical modifications. For phosphoproteomics, P‐CNCs‐Ti^4^^+^ enriched 5,225 phosphopeptides from mouse liver tissue, outperforming commercial TiO_2_ and identifying over 100 of 3‐phosphoglyceryl modifications in glycolytic enzymes. Comprehensive life cycle assessment demonstrates the environmental sustainability of this approach, achieving a 76% cost reduction compared to commercial materials while significantly lowering the associated environmental footprint. This work pioneers P‐CNCs as a sustainable, high‐performance platform for multiplexed PTM profiling, bridging glycoproteomics and phosphoproteomics with broad applications in biomarker discovery and metabolic pathway analysis.

## Graphical abstract

AAA

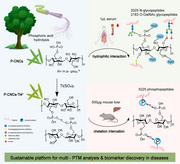

## 1 Introduction

Protein post‐translational modifications (PTMs) are crucial cellular mechanisms that modulate various protein properties, including folding, spatial distribution, turnover, and functions [1, 2]. Among the many types of PTMs, protein glycosylation and phosphorylation are two of the most prominent, significantly influencing numerous biological processes and diseases. Glycosylation demonstrates remarkable structural and functional diversity [3, 4]. This complexity arises from the combinatorial assembly of the ten common monosaccharides into thousands of distinct glycans, driven by variations in linkage positions, anomeric configurations, and branched topologies, as well as other potential chemical modifications. Such diversity critically regulates protein folding, stability, intracellular trafficking, and biological activity [5, 6]. In contrast, phosphorylation is a reversible enzymatic reaction regulated by kinases, occurring in approximately 30% of all eukaryotic proteins, primarily on serine, threonine, and tyrosine residues [7]. Phosphorylation plays vital roles in cell division, signal transduction, gene expression regulation, and protein interactions [8]. Therefore, accurate monitoring of these PTMs is critical for understanding pathogenesis and facilitating biomarker discovery.

Mass spectrometry (MS) has emerged as a powerful tool for providing extensive information on proteins and peptides at a large scale [9, 10, 11]. However, the high abundance of non‐modified peptides and low ionization efficiency necessitate effective enrichment of phosphopeptides and glycopeptides prior to MS analysis [12, 13]. Several strategies, including hydrophilic interaction liquid chromatography (HILIC) [14, 15], lectin affinity chromatography [16], covalent interactions [17], and chemo‐enzymatic chemistry [18], have been adopted to enrich intact glycopeptides. Among these, HILIC has gained popularity due to its ability to selectively enrich intact N‐linked and O‐GalNAc glycopeptides without compromising their structural integrity [19]. For phosphopeptide enrichment, strategies such as immobilized metal ion affinity chromatography (IMAC) [20], metal oxide affinity chromatography (MOAC) [21], immunoprecipitation, and phosphorylation derivation have been employed, with IMAC being the most widely adopted due to its exceptional specificity arising from the strong affinity between phosphate groups and metal ions [22].

Despite their effectiveness, the preparation processes for these enrichment materials often involve tedious, environmentally unfriendly, and costly methods, making them unsuitable for large‐scale sample processing [23, 24]. Recently, materials adhering to “green chemistry” principles have gained attention in various fields, including catalysis, energy storage, water purification, chromatography, and biosensors etc [25, 26]. Several natural hydrophilic materials, particularly polysaccharides such as cotton, agarose, and chitosan, have been functionally modified for use as enrichment materials [27, 28, 29]. However, the inherent instability of these natural polysaccharides in acidic or alkaline conditions poses significant challenges due to their poor selectivity and low durability [30]. Cellulose nanocrystals (CNCs), rod‐like nanostructures derived from the natural breakdown of cellulose polymer found in the cell walls of plants, algae, and some bacteria, offer a promising alternative [31, 32]. CNCs possess a high aspect ratio and consist of glucose units linked by β (1→4) ‐glycosidic bonds, with each unit featuring multiple hydroxyl groups. As sustainable materials derived from renewable sources, CNCs are valued for their unique mechanical, thermal, and optical properties [33, 34, 35].

Herein, we developed a simplified one‐step method for preparing phosphorylated cellulose nanocrystals (P‐CNCs) via phosphoric acid hydrolysis. The P‐CNCs feature abundant hydroxyl groups and a high transverse‐to‐vertical ratio, enabling highly effective enrichment of intact glycopeptides, including both N‐linked and O‐GalNAc glycosylation. Additionally, the introduced phosphate groups in P‐CNCs serve as direct Ti^4^^+^ chelation sites for the preparation Ti^4^^+^ immobilized P‐CNCs (P‐CNCs‐Ti^4+^), which could be used for phosphopeptide enrichment. The enhanced rigidity of P‐CNCs ensures stability under acidic and basic conditions, which are typical in enrichment experiments. This method provides a more efficient, convenient, and environmentally friendly alternative to conventional glycopeptide and phosphopeptide enrichment techniques.

## 2 Results and Discussion

### 2.1 Preparation and Characterization of P‐CNCs and P‐CNCs‐Ti^4+^

**SCHEME 1:**
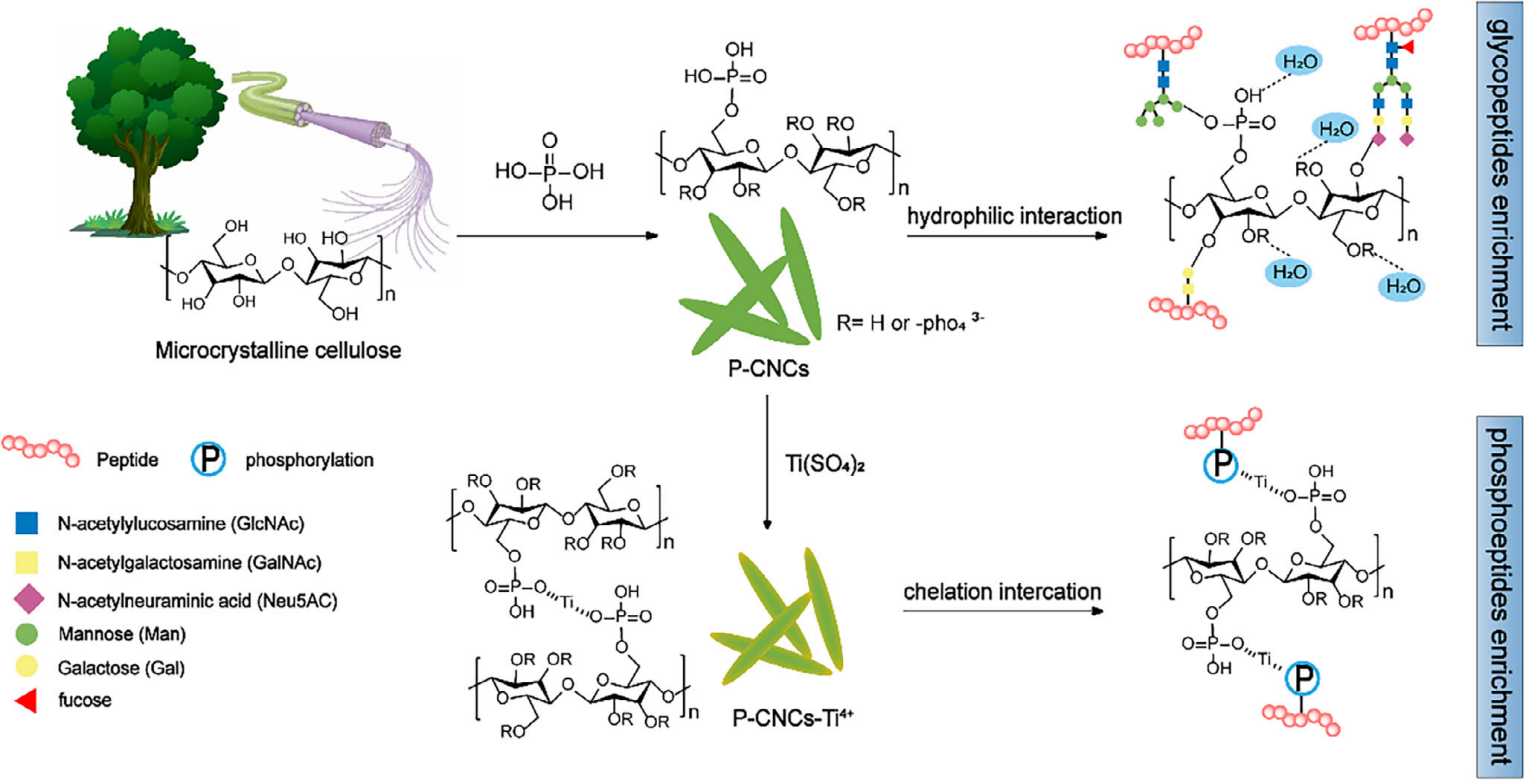
Facile preparation and dual‐affinity enrichment strategy using phosphorylated cellulose nanocrystals (P‐CNCs). One‐step phosphoric acid hydrolysis converts microcrystalline cellulose (MCC) into P‐CNCs bearing abundant surface hydroxyl and phosphate groups. P‐CNCs directly enrich glycopeptides via HILIC mode, while Ti^4^^+^ chelation yields P‐CNCs‐Ti^4+^ for selective phosphopeptide enrichment via IMAC.

CNCs are environmentally benign and hydroxyl‐rich green nanomaterials with biocompatibility and tunability, ideal for HILIC materials and affinity ligand matrices. In this study, the P‐CNCs were synthesized via a one‐step in situ hydrolysis procedure with phosphoric acid (Scheme 1). The resulting material enables direct enrichment of intact glycopeptides through HILIC interactions. Furthermore, Ti^4^^+^ ions were immobilized via affinity interactions on phosphate groups introduced during synthesis, facilitating phosphopeptide enrichment. Additionally, multi‐hydroxyl groups suppress non‐specific adsorption of non‐phosphopeptides.

**FIGURE 1:**
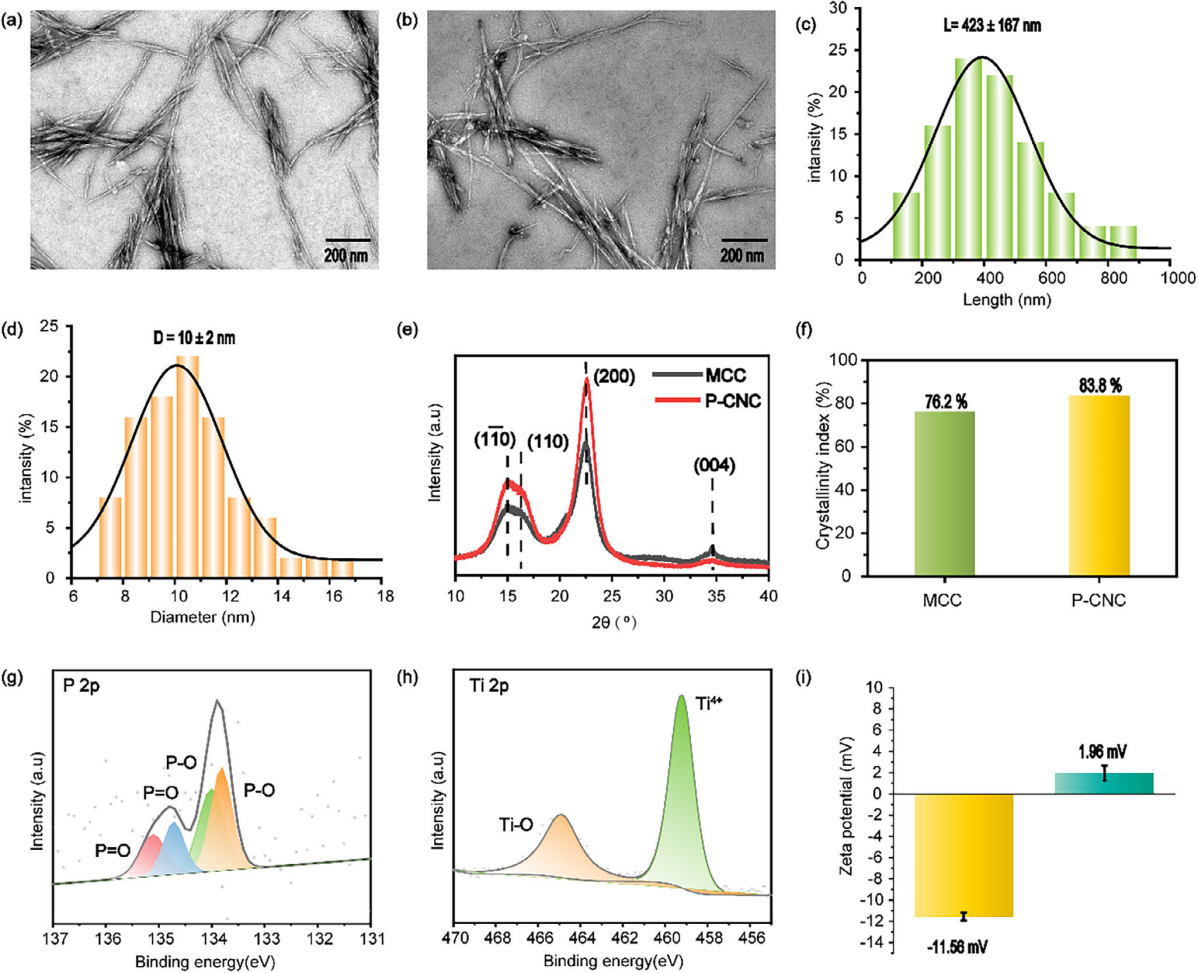
Structural and surface characterization of P‐CNCs and P‐CNCs‐Ti^4^^+^. Transmission electron microscopy (TEM) images of P‐CNCs (a) and P‐CNCs‐Ti^4+^ (b); The size distribution histograms of P‐CNCs showing length (c) and diameter (d); (e) X‐ray diffraction (XRD) patterns of P‐CNCs before and after Ti^4^^+^ coordination; (f) Corresponding crystallinity indices; High‐resolution X‐ray photoelectron spectroscopy (XPS) spectra of P‐CNCs‐Ti^4+^ showing the P 2p (g) and Ti 2p (h) regions; (i) Zeta potential measurements of P‐CNCs (left) and P‐CNCs‐Ti^4+^ (right).

P‐CNCs and P‐CNCs‐Ti^4+^ were characterized by transmission electron microscopy (TEM). Both materials exhibited well‐dispersed short‐rod morphologies with narrow size distributions (Figure 1a,b). The P‐CNCs showed an average length of 423 ± 167 nm and a diameter of 10 ± 2 nm (Figure 1c,d). After Ti^4+^ immobilization, the nanocrystals retained their original morphology and dispersibility, with comparable dimensions of 417 ± 169 nm in length and 10 ± 2 nm in diameter (Figure S1), indicating that Ti^4^^+^ coordination did not induce structural aggregation or morphological degradation. X‐ray diffraction (XRD) analysis confirmed the crystallinity of both materials after hydrolysis, revealing characteristic cellulose peaks at 2θ = 15.3° ($1\bar{1}0$), 16.7° (110), 22.4° (200), and 34.8° (004). Notably, the crystallinity of P‐CNCs (83.8%) exceeded that of microcrystalline cellulose (MCC, 76.2%) (Figure 1e,f). TEM characterization further showed that P‐CNCs retained their morphology across a wide pH range (2‐12) (Figure S2), indicating that removal of amorphous regions ensures their stability under enrichment‐relevant conditions. X‐ray photoelectron spectroscopy (XPS) analysis of P‐CNCs‐Ti^4+^ revealed characteristic peaks for C (297.9 eV), O (544.9 eV), P (143.9 eV), and Ti (474.9 eV) in the full survey spectrum (Figure S3). High‐resolution P2p spectra displayed two peaks at 133.9 (P─O) and 134.8 eV (P═O), while the Ti2p region showed peaks at 459.2 eV (Ti^4+^) and 464.9 eV (Ti─O) (Figure 1g,h). Zeta potential measurements demonstrated a significant shift from ‐11.56 mV for P‐CNCs to +1.96 mV for P‐CNCs‐Ti^4+^, confirming the successful chelation of Ti^4^^+^ ions by the phosphate group (Figure 1i). These results demonstrate the successful functionalization of cellulose nanocrystals with phosphate groups and titanium ions, supporting their suitability for glycopeptide and phosphopeptide enrichment.

### 2.2 Evaluation of P‐CNCs and P‐CNCs‐Ti^4+^ for Selective Enrichment of Intact Glycopeptides and Phosphopeptides

**FIGURE 2:**
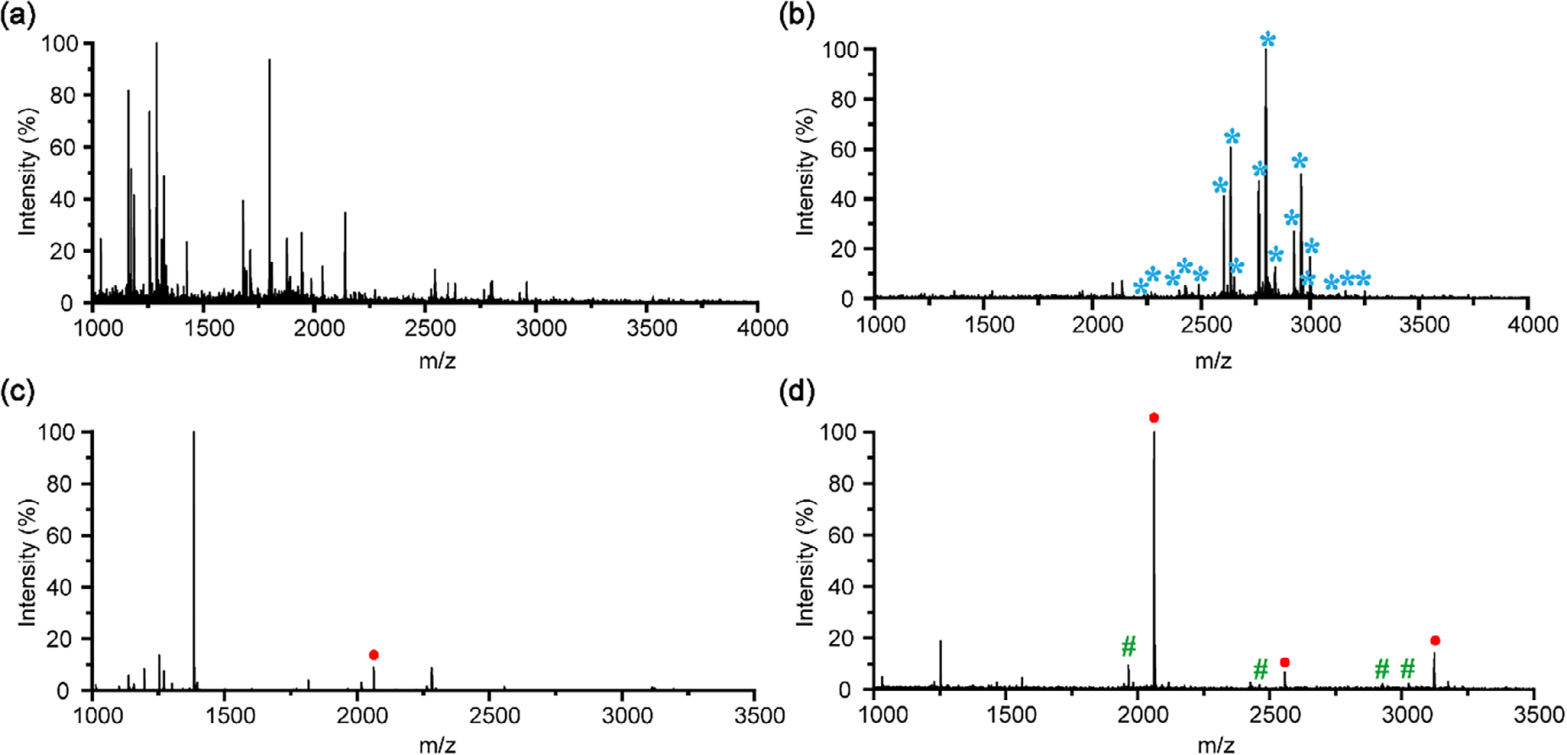
Matrix‐Assisted Laser Desorption/ Ionization Time of Flight Mass Spectrometry (MALDI‐TOF MS) spectra of tryptic digest from human IgG and β‐casein before and after enrichment with P‐CNCs and P‐CNCs‐Ti^4+^. (a) Direct analysis of IgG tryptic digest and (b) analysis after enrichment with P‐CNCs; (c) direct analysis of β‐casein tryptic digest and (d) analysis after enrichment with P‐CNCs‐Ti^4+^; (^*^, N‐linked intact glycopeptide; ●, phosphopeptides; #, de‐phosphopeptides).

HILIC has become a widely adopted approach for intact glycopeptide enrichment, based on the fundamental principle of preferential glycopeptide retention on hydrophilic stationary phases [36, 37]. The abundant surface hydroxyl groups of P‐CNCs endow them with exceptional hydrophilicity, making them particularly promising for glycopeptide enrichment. To assess the enrichment performance, the P‐CNCs were utilized to enrich N‐linked intact glycopeptides from the tryptic digests of human IgG (a model glycoprotein). Systematic optimization revealed the critical influence of acetonitrile concentration in the loading buffer. As shown in Figure S4, some of the glycopeptides were successfully captured at 75% acetonitrile (ACN), while there were still significant portions of glycopeptides missed. Increasing the acetonitrile proportion to 78% significantly enhanced glycopeptide recovery while simultaneously suppressing non‐glycopeptide signals. Further elevating the acetonitrile did not yield additional gains in glycopeptide enrichment and instead promoted non‐specific binding of non‐glycopeptides. Through further careful optimization of elution conditions, an optimal protocol was established for the intact glycopeptide enrichment using loading buffer: ACN/H_2_O/trifluoroacetic acid (TFA) (78:21:1, v/v/v) and elution solvent: ACN/H_2_O/ formic acid (FA) (30:69:1, v/v/v) (Figure S5). As shown in Figure 2a,b, the non‐glycopeptide signals were prominent before enrichment of the IgG enzymatic digest by Matrix‐Assisted Laser Desorption/ Ionization Time of Flight Mass Spectrometry (MALDI‐TOF MS), while the peaks of the intact glycopeptides were clearly observed with almost no interference of non‐glycopeptide signals after P‐CNCs based enrichment (Table S1).

Correspondingly, the conditions for phosphopeptide enrichment using P‐CNCs‐Ti^4+^ materials were evaluated, employing ACN/H_2_O/TFA (80/17/3, v/v/v) as the loading buffer and 10% ammonia as the elution buffer (Figure S6). MALDI‐TOF MS analysis revealed that direct analysis of the β‐casein tryptic digest detected only one mono‐phosphopeptide (FQEEQQQTEDELQDK). In contrast, all three phosphopeptides, including two multi‐phosphopeptides (FQEEQQQTEDELQDK, FQEEQQQTEDELQDKIHPF, RELEELNVPG EIVELEESITR), were clearly detected after enrichment with P‐CNCs‐Ti^4+^ materials (Figure 2c,d; Table S2). These results indicated that P‐CNCs and P‐CNCs‐Ti^4+^ effectively enrich intact glycopeptides and phosphopeptides, respectively, highlighting their potential for glycoproteomic and phosphoproteomic applications.

### 2.3 Comprehensive Profiling of N‐Linked Glycoproteomes in Human Serum Using P‐CNCs for Enhanced Glycopeptide Enrichment

N‐linked glycosylation, a crucial post‐translational modification process for proteins, plays a significant role in the onset and progression of numerous diseases [38, 39]. Site‐specific analysis of N‐linked glycosylation in serum can help identify disease‐specific biomarkers to aid diagnosis, severity assessment, and progression monitoring. Based on the high specificity of glycopeptide enrichment, the P‐CNCs materials were further applied to the enrichment and identification of N‐linked intact glycopeptides in serum.

**FIGURE 3:**
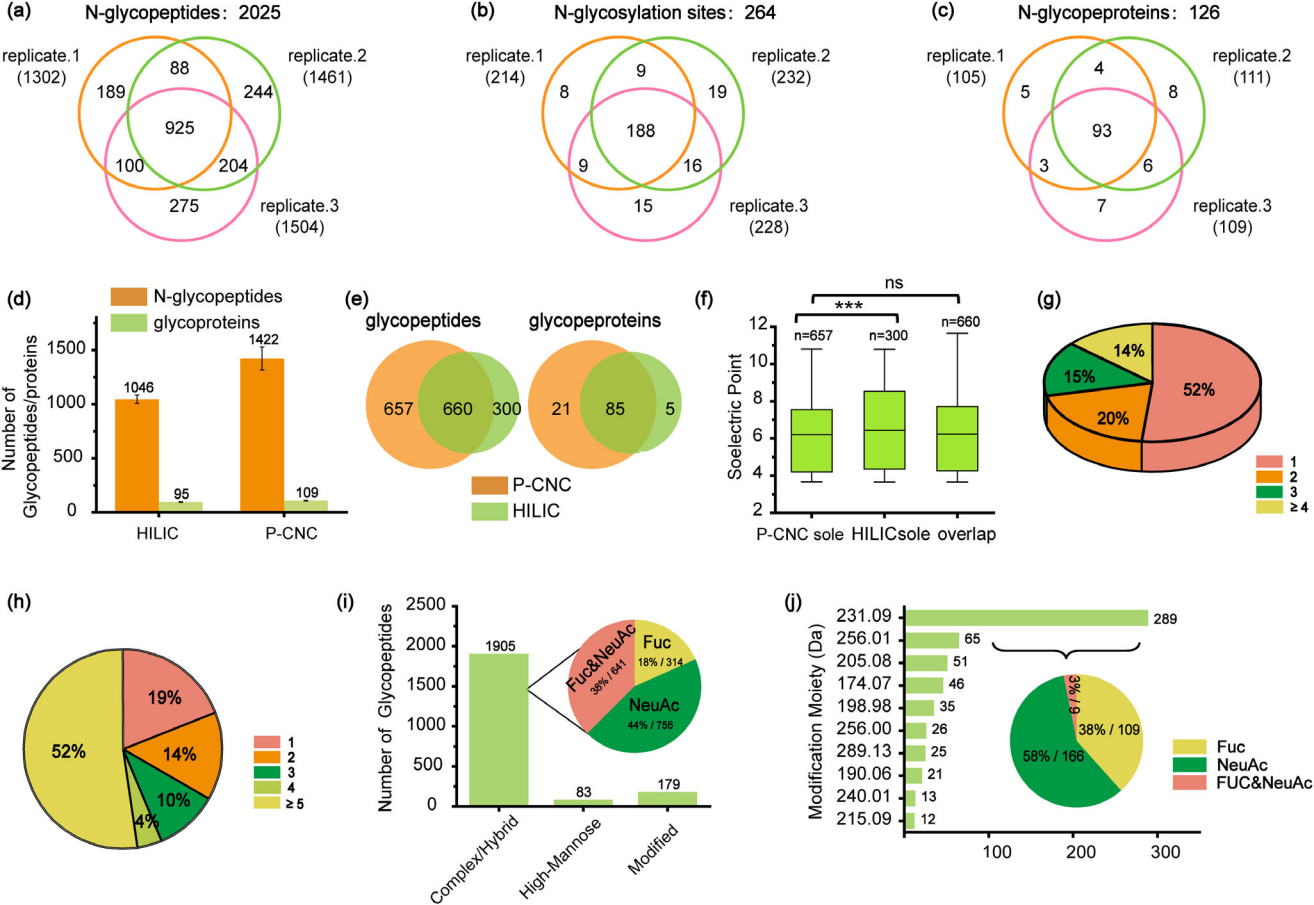
Comprehensive N‐glycoproteome profiling of human serum using P‐CNCs enrichment. Venn diagrams of identified (a) intact N‐glycopeptides, (b) N‐glycosylation sites and (c) N‐glycoproteins in three technical replicates; (d) Comparison of the numbers of identified N‐glycopeptides and N‐glycoproteins between HILIC and P‐CNCs methods; (e) Overlap of identified N‐glycopeptides and N‐glycoproteins between HILIC and P‐CNCs approaches; (f) Distribution of isoelectric points (pI) of N‐glycopeptides enriched by HILIC and P‐CNCs (^***^, *p* < 0.001; ns, not significant); (g) Frequency distribution of N‐glycosylation sites per glycoprotein; (h) Distribution of site‐specific glycans per glycoprotein; (i) Relative abundance of different N‐glycan types; (j) Mass distribution of the top ten N‐glycan compositions.

As illustrated in Figure 3a–c, 1302, 1461, and 1504 intact glycopeptides were identified from just 1 µL of serum tryptic digest in three technical replicates, corresponding to 214, 232, and 228 glycosites in 105, 111, and 109 glycoproteins, respectively. In total, 2025 unique N‐linked glycopeptides were identified, corresponding to 264 N‐glycosylation sites in 126 proteins, with excellent reproducibility (Data S1). Motif analysis of the identified N‐glycosylation sites revealed a strong enrichment of the canonical Asn–X–Ser/Thr (X ≠ Pro) consensus sequence, supporting the reliability of site‐specific assignments (Figure S7). Compared to maltose‐based enrichment, P‐CNCs increased the number of identified glycopeptides by more than 40% (Figure 3d), capturing 70% of maltose‐detected glycopeptides identified in at least two of the three technique replicates (Figure 3e; Data S2). In addition, 657 intact glycopeptides were exclusively identified using the P‐CNC‐based method, highlighting the complementarity between the two enrichment strategies. Notably, glycopeptides enriched by P‐CNCs exhibited a shift toward lower isoelectric point (pI) values compared to those enriched by the maltose‐based method (Figure 3f). This trend is attributable to the combined hydrophilicity and weak negative surface charge of P‐CNCs, which facilitates stronger interactions with glycopeptides than those achievable using neutral maltose‐based materials. Protein N‐linked glycosylation exhibits intrinsic heterogeneity at both the protein level, reflected by multiple glycosylation sites per glycoprotein, and the site level, manifested as diverse glycoforms at individual sites, as revealed by the P‐CNC‐based serum analysis. Approximately 50% of glycoproteins were identified with a single glycosylation site, whereas only about 29% of glycoproteins contained more than two glycosylation sites (Figure 3g). In contrast, individual glycosylation sites exhibited substantially greater structural complexity. Only 19% of glycosylation sites carried a single glycan, whereas approximately 50% were modified by more than five distinct glycoforms (Figure 3h). In addition, approximately 90% of the identified glycopeptides were classified as complex/hybrid‐type glycans, while fewer than 5% corresponded to high‐mannose structures (Figure 3i). Among the complex/hybrid‐type glycopeptides, 82% were sialylated, 56% were fucosylated, and 38% carried both modifications. Intriguingly, we also identified 179 intact glycopeptides with unknown modifications of glycans, and most of them with a mass shift of +231.09 Da (Figure 3j). Further analysis indicated that the unknown modification of +231.09 Da was predominantly attached to the high mannose type glycans. Collectively, these results indicate P‐CNCs as a highly effective enrichment material for N‐linked intact glycopeptides, permitting detailed mapping of glycosylation patterns, as well as rare modifications, with significant implications for glycoproteomic research in complex biological samples.

**FIGURE 4:**
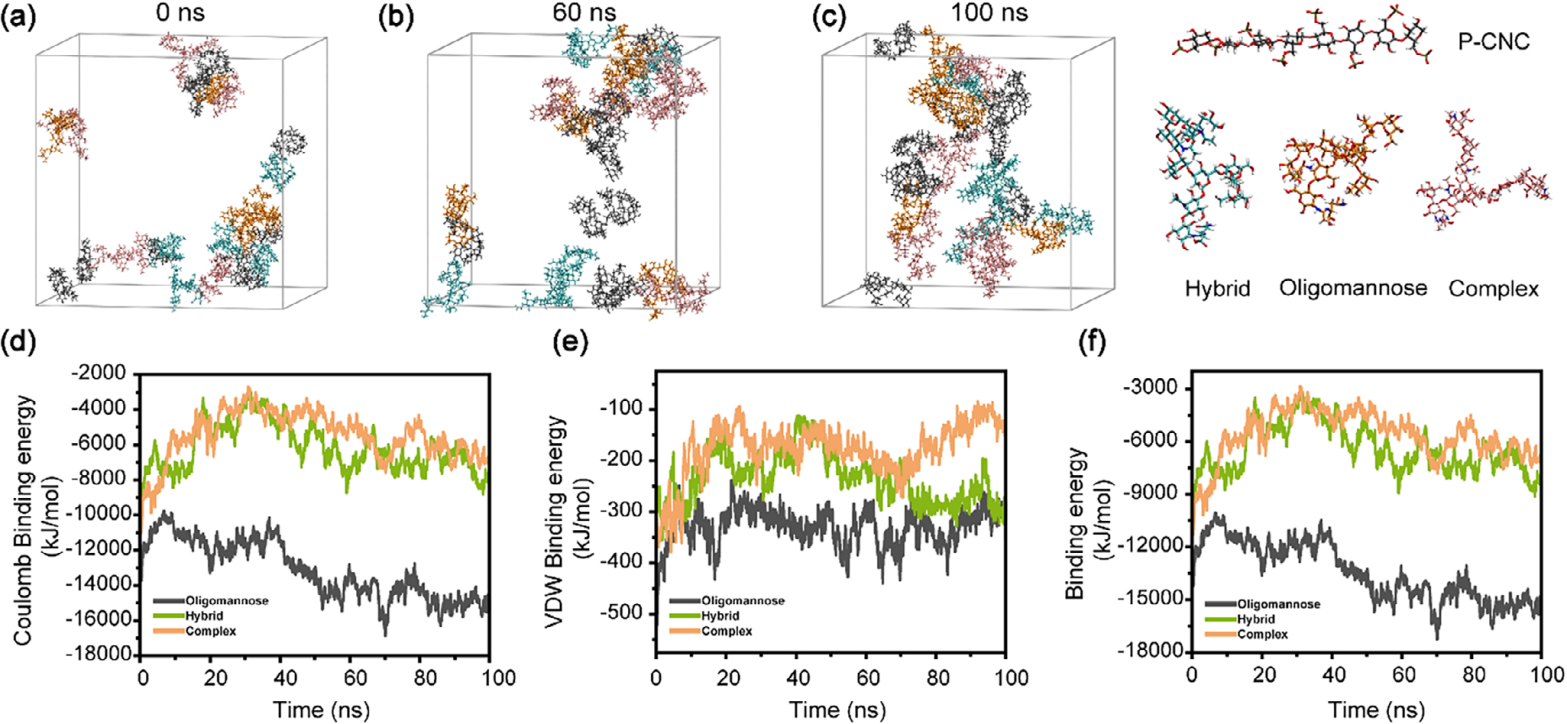
Molecular dynamics (MD) analysis of N‐Glycopeptide adsorption onto P‐CNCs. Conformational evolution of representative P‐CNCs/N‐glycopeptide complexes during 100‐ns simulations: (a) Initial binding at 0 ns, (b) intermediate reorientation at 60 ns, and (c) stable adsorption at 100 ns; Energy decomposition analysis of N‐glycopeptide adsorption on P‐CNCs: (d) Coulomb Binding energy, (e) van der Waals (VDW) Binding energy, and (f) total Binding energy.

To elucidate the molecular mechanism governing N‐glycopeptide enrichment by P‐CNCs, all‐atom molecular dynamics (MD) simulations were performed using GROMACS 2023.5 with the GAFF2 force field. The simulations revealed favorable interactions between P‐CNCs and N‐glycopeptides, as reflected by binding free energy calculations. Trajectory analysis showed that the glycopeptide P‐CNC interactions approached a stable state within approximately 60 ns, supporting the effective capture capability of P‐CNCs (Figure 4a–c). Energy decomposition further revealed that Coulombic interactions dominated the attractive forces (>90% of total binding energy), substantially outweighing van der Waals contributions.

The primary source of Coulombic attraction stems from synergistic hydrogen‐bonding networks and electrostatic fields generated by surface phosphate groups (−PO_4_^3^^−^) and hydroxyl groups (─OH) on P‐CNCs (Figure 4d). In contrast, van der Waals (VDW) interactions arise from optimal spatial matching, characterized by an approximately 0.25 nm spacing between surface hydroxyl groups, which facilitates glycopeptide adsorption (Figure 4e; Figure S8). Further comparison of binding affinities for three representative N‐glycan types, including oligomannose, complex, and hybrid glycans, suggested stronger simulated interactions for oligomannose‐type glycopeptides relative to the other two glycoforms (Figure 4f). This finding shows a significant discrepancy from experimental observations, which indicate that complex and hybrid glycans (∼ 88%) predominate, while oligomannose‐type glycans (∼ 4%) are less abundant. It is hypothesized that this may arise from the low abundance of oligomannose‐type glycans in actual serum samples [40, 41]. Notably, elevated oligomannose‐type glycan levels have been documented in pathological conditions [42, 43], underscoring the potential utility of P‐CNCs in detecting disease‐associated glycomic alterations.

### 2.4 In‐depth O‐Glycoproteome Analysis Reveals Site‐Specific Fucosylation Patterns Through P‐CNCs‐Based Enrichment

O‐GalNAc is another type of protein glycosylation, involving the attachment of N‐acetylgalactosamine (GalNAc) to the sugar chain of proteins. It plays crucial roles in various biological processes, including cell adhesion, migration, apoptosis, inflammatory response, immune evasion, and viral infection [44, 45]. Additionally, O‐GalNAc is closely related to the pathogenesis of numerous diseases, such as microbial infections, tumors, autoimmune diseases, metabolic diseases, cardiovascular diseases, and neurodegenerative diseases [46]. Compared to N‐linked glycopeptides, the identification of O‐glycopeptides remains a more challenging task due to the latter's inherently lower abundance, lack of conserved modification sequence motifs, and complex microstructure of their glycans. Leveraging the successful enrichment of N‐linked glycopeptides, we further utilized P‐CNCs material to enrich O‐GalNAc glycopeptides, which typically possess shorter sugar chains and weaker hydrophilicity. To minimize interference from N‐ glycosylation, human serum samples were treated with PNGase F to remove N‐glycans prior to trypsin digestion.

**FIGURE 5:**
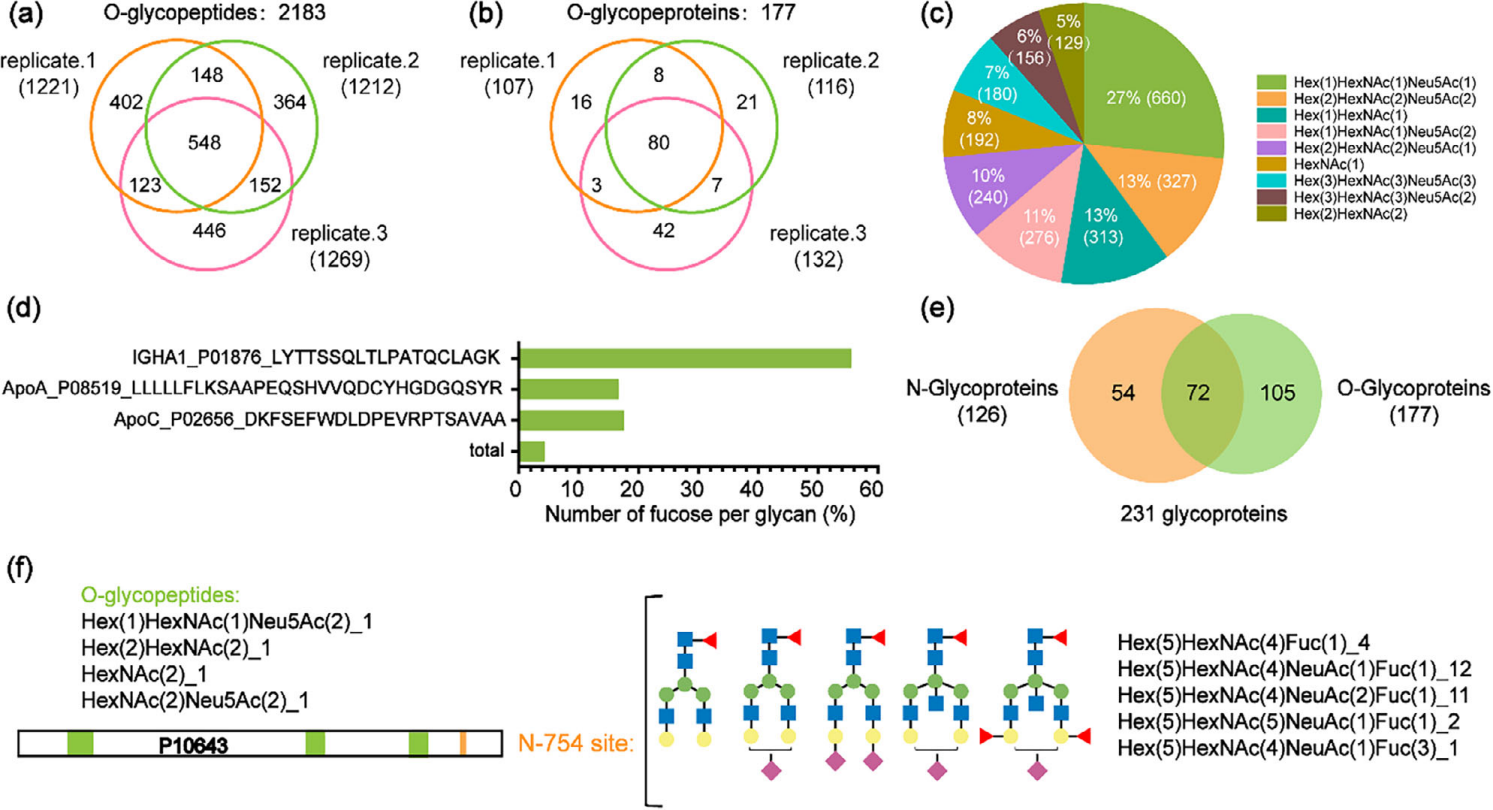
Comprehensive O‐glycoproteome analysis of human serum after P‐CNCs enrichment. Venn diagram of identified (a) O‐glycopeptides and (b) O‐glycoproteins in three technical replicates; (c) Distributions of O‐GalNAc glycoforms in human serum protein; (d) Distribution of fucosylation levels on O‐glycans; (e) Overlap of identified N‐glycoproteins and O‐glycoproteins using P‐CNCs; (f) Site‐specific map of identified N‐linked and O‐GalNAc glycans on complement component C7 (C7, P10643).

As shown in Figure 5a,b, 1,221, 1,212, and 1,269 O‐GalNAc glycopeptides were identified from three technical replicates of enzyme‐digested samples, using just 1 µL of serum tryptic digest, corresponding to 107, 116, and 132 glycoproteins, respectively (Data S3). Glycan composition analysis revealed that O‐glycans were predominantly composed of (Hex)_1_(HexNAc)_1_(Neu5Ac)_1_, (Hex)_1_(HexNAc)_1_, and (Hex)_2_(HexNAc)_2_(Neu5Ac)_2_ moieties, collectively accounting for approximately 50% of the total glycopeptide population (Figure 5c). This distribution is consistent with previous reports of glycopeptides enriched using other hydrophilic materials [47]. By contrast, core fucosylation in N‐glycosylation has been extensively investigated within cancer research, exemplified by the enhanced diagnostic accuracy of alpha fetoprotein (AFP) core fucosylation for hepatocellular carcinoma. In contrast, site‐specific fucosylation of O‐GalNAc glycans at the intact glycopeptide level remains poorly characterized [48]. A total of 7,931 O‐GalNAc glycopeptide‐spectrum matches (PSMs) were identified. When focusing on O‐GalNAc glycans with simple compositions (≤ 3 HexNAc and Hex residues), 347 PSMs (approximately 5%) were assigned to fucosylated O‐GalNAc glycopeptides. Further analysis revealed that these 347 PSMs corresponded to 128 fucosylated glycopeptides, representing 117 unique peptide sequences derived from 40 proteins, with variable degrees of fucosylation occupancy. Relatively high fucosylation occupancy was observed on glycopeptides from immunoglobulin heavy constant alpha 1 (IGHA1), which mediates antigen clearance [49, 50]. As shown in Figure 5d, more than 50% of glycopeptides corresponding to the IGHA1 sequence (LYTTSSQLTLPATQCLAGK) were fucosylated. Fucosylation was also detected on apolipoprotein C‐III (ApoC‐III), which is secreted by the liver and intestine and is associated with triglyceride‐rich lipoproteins. ApoC‐III contains an O‐GalNAc site at Thr94 [51]. Using P‐CNCs enrichment, six distinct glycan compositions were identified at this site, three of which were fucosylated. The preferential fucosylation observed on specific proteins such as IGHA1 and ApoC‐III suggests a potential functional relevance of O‐GalNAc fucosylation, providing a basis for future studies of protein regulation and disease mechanisms.

A simultaneous and in‐depth analysis of N‐linked and O‐GalNAc glycosylation was performed using human serum tryptic digests. A total of 177 O‐glycoproteins and 126 N‐glycoproteins were identified, with 72 proteins carrying both types of glycosylation (Figure 5e). Gene ontology (GO) analysis revealed that the identified glycoproteins were primarily involved in complement activation, humoral immune response, and negative regulation of hemostasis (Figure S9). Notably, complement component C7 (C7), which serves as a membrane anchor for the membrane attack complex (MAC) and plays a key role in innate and adaptive immunity by forming pores in target cell membranes, was found to carry both N‐linked and O‐GalNAc glycosylation. Using the P‐CNCs enrichment method, five N‐linked intact glycopeptides at Asn754 and four O‐GalNAc glycopeptides were identified (Figure 5f). While all identified N‐glycopeptides were core‐fucosylated, no fucosylation was observed on the O‐GalNAc glycopeptides, enabling site‐specific glycosylation mapping at the intact glycopeptide level.

### 2.5 P‐CNCs‐Ti^4+^ Enables Comprehensive Phosphoproteome Analysis and Discovery of Non‐Canonical Phosphorylated Modifications

Besides glycosylation, phosphorylation is another important type of post‐translational protein modification. Currently, the enrichment strategies for phosphorylation primarily include metal ion chelation and metal oxidation affinity chromatography, both of which require tedious preparation steps. Achieving one‐step preparation of materials remains the key step for phosphopeptide enrichment. Collectively, these results indicate that P‐CNCs enable efficient glycopeptide enrichment via phosphate‐group‐enhanced hydrophilic interactions and that the same phosphate functionalities also serve as metal‐chelating ligands for phosphopeptide enrichment.

**FIGURE 6:**
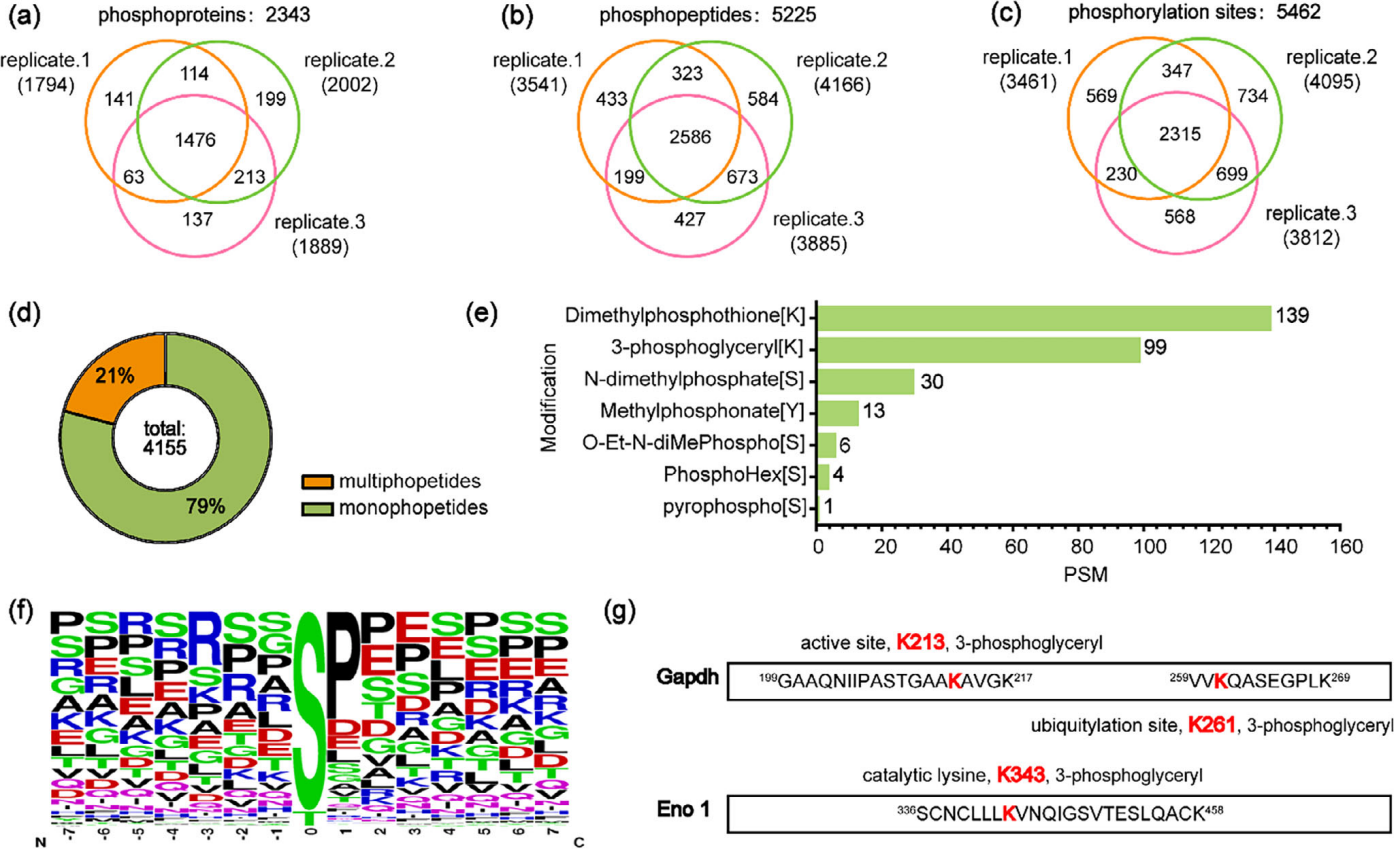
Comprehensive phosphoproteome analysis of mouse liver using P‐CNCs–Ti^4^^+^ enrichment. Venn diagrams of identified (a) phosphoproteins, (b) phosphopeptides and (c) phosphorylation sites in three technical replicates; (d) Distribution of mono‐ and multi‐phosphopeptides identified on phosphoproteins; (e) Peptide‐spectrum matches (PSMs) corresponding to non‐canonical phosphorylation modifications; (f) Motif analysis of identified phosphopeptides; (g) Site‐specific identification of 3‐phosphoglyceryl [K] modifications on glyceraldehyde‐3‐phosphate dehydrogenase (GAPDH) and α‐enolase (ENO1).

The P‐CNCs‐Ti^4+^ demonstrated efficient enrichment of phosphopeptides from the model protein of β‐casein, and were further utilized for the analysis of protein phosphorylation in complex biological samples from mouse liver tissues. The enriched phosphopeptides were then subjected to LC‐MS/MS analysis. This analysis yielded the unambiguous identification of 5,225 unique phosphopeptides, which corresponded to 5,462 distinct phosphorylation sites on 2,343 phosphorylated proteins in three technical replicates (Figure 6a–c; Data S4). It exhibits more significant enrichment properties than other materials. In particular, compared to the commercial TiO_2_ kit, the P‐CNCs‐Ti^4^^+^ materials enabled the identification of over twice as many phosphopeptides, demonstrating their superior enrichment efficiency (Table S3) [52]. Moreover, more than 70% of these phosphopeptides were identified in at least two of the three technical replicates, demonstrating high reproducibility of the enrichment method using P‐CNCs‐Ti^4+^ materials. Analysis of the phosphorylation site distribution revealed that 3,282 phosphopeptides (79%) were mono‐phosphorylated, each containing a single phosphorylation site, whereas 873 phosphopeptides (21%) were multi‐phosphorylated, containing two or more phosphorylation sites (Figure 6d). Motif analysis demonstrated that phosphorylation predominantly occurred at serine or threonine residues, typically followed by proline at the adjacent position (Figure 6f), consistent with established phosphorylation patterns reported in previous studies [53, 54].

Beyond classical protein phosphorylation, numerous non‐canonical phosphorylated modifications remain insufficiently characterized and are often overlooked in conventional proteomic analyses [55]. To systematically profile diverse phosphorylated forms, an open‐search strategy was further applied to peptides enriched using P‐CNCs–Ti^4^^+^ materials. In addition to the conventional phosphorylation, we identified seven types of atypical phosphorylated modifications, including Dimethylphosphothione [K], 3‐phosphoglyceryl [K], N‐dimethylphosphate [S], Methylphosphonate [Y], O‐Et‐N‐diMePhospho [S], PhosphoHex [S], and pyrophospho [S]. The spectra were interpreted by setting the seven phosphorylated modifications as variable modifications, and all were identified with a false discovery rate (FDR) below 1% (Figure 6e). Among these non‐canonical modifications, it was found that Dimethylphosphothione [K] and 3‐phosphoglyceryl [K] were the top two modifications (Data S5). Besides, the 3‐phosphoglyceryl [K] modification, resulting from covalent attachment of 1,3‐diphosphoglycerate (1,3‐BPG) to lysine residues, has previously been implicated in the regulation of glycolytic pathways [56]. Unambiguous modification sites were identified on multiple glycolytic enzymes, supporting the robustness and reliability of the detected phosphorylation events. Notably, 3‐phosphoglyceryl [K] modifications were identified at both the catalytic active site (K213) and a known ubiquitylation site (K261) of glyceraldehyde‐3‐phosphate dehydrogenase (GAPDH). In addition, the catalytic lysine residue (K343) of α‐enolase (ENO1), which catalyzes the conversion of 2‐phosphoglycerate to phosphoenolpyruvate, was also identified as being modified by 3‐phosphoglyceryl [K], a modification that has the potential to impair ENO1 enzymatic activity (Figure 6g; Figure S10). Taken together, these results demonstrate that the P‐CNCs–Ti^4^^+^‐based phosphopeptide enrichment strategy not only offers practical advantages, including facile material preparation and high enrichment efficiency, but also enables comprehensive profiling of diverse phosphorylation‐related post‐translational modifications.

### 2.6 Sustainability and Cost‐Effectiveness of the P‐CNCs‐Ti^4+^ Enrichment Platform

**FIGURE 7:**
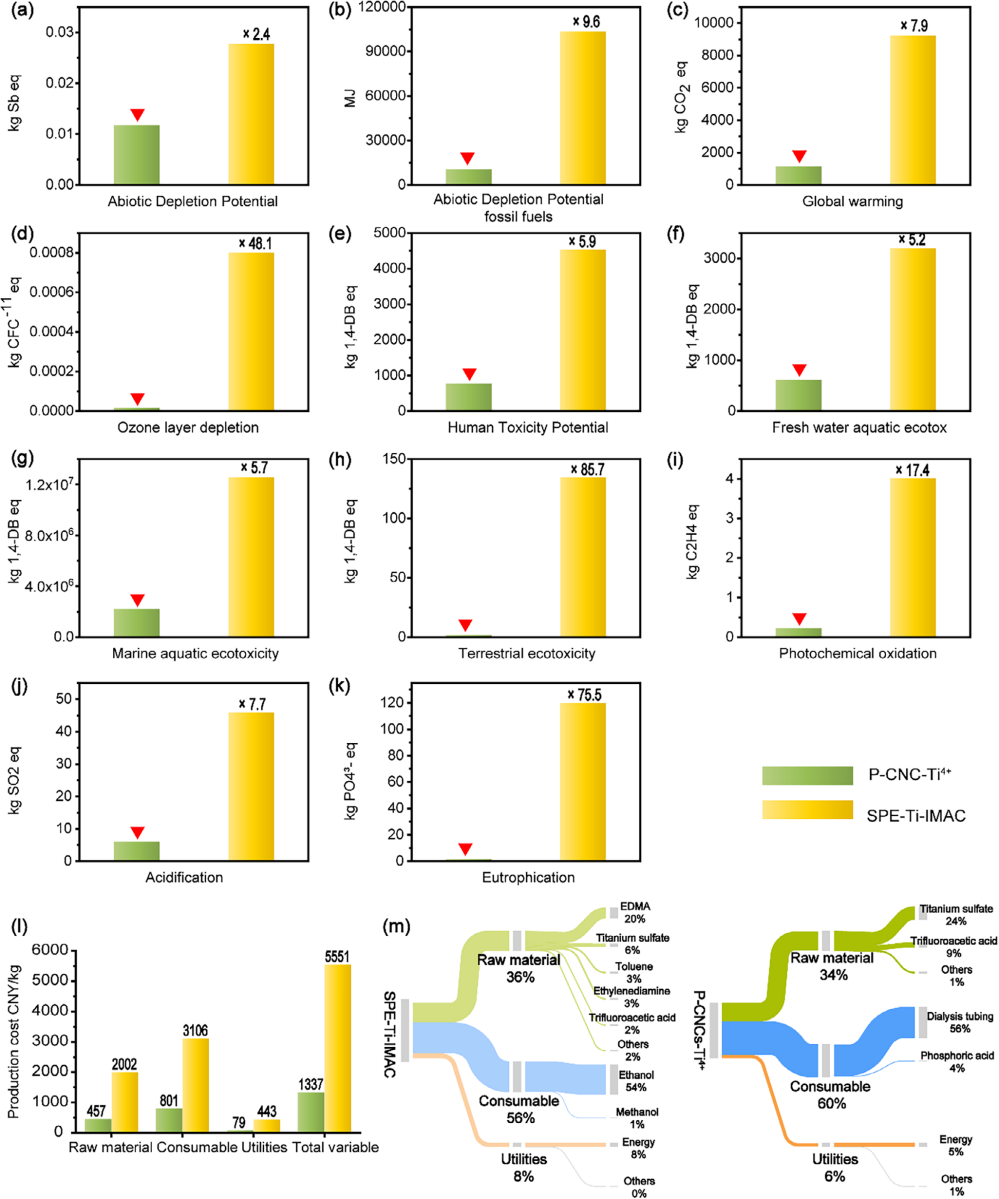
Comparative Life cycle assessment (LCA) and cost analysis of SPE–Ti–IMAC and P‐CNCs‐Ti^4+^ materials. LCA assessment results of environmental impact categories: (a) abiotic depletion potential, (b) abiotic depletion potential fossil fuels, (c) global warming, (d) ozone layer depletion, (e) human toxicity potential, (f) fresh water aquatic ecotox, (g) marine aquatic ecotoxicity, (h) terrestrial ecotoxicity, (i) photochemical oxidation, (j) acidification, and (k) eutrophication; Detailed production cost analysis of SPE‐Ti‐IMAC and P‐CNCs‐Ti^4+^: (l) comparative analysis results of material preparation cost, (m) cost composition of material preparation.

To evaluate the sustainability of the dual‐functional enrichment platform, a cradle‐to‐gate life cycle assessment (LCA) was conducted for P‐CNCs‐Ti^4+^ materials, encompassing the entire production process, from MCC sourcing to Ti^4+^ chelation. The CML‐IA baseline methodology (v3.08) was employed to quantify environmental impacts. Strikingly, P‐CNCs‐Ti^4+^ demonstrates significantly lower impacts than commercial Immobilized Ti^4+^ affinity chromatography microspheres for solid‐phase extraction (SPE‐Ti‐IMAC) microspheres across all assessed categories (Figure 7a–k) [57, 58]. Importantly, the main sources of pollution in the production process of P‐CNCs are electricity and phosphoric acid, while the environmental impact of cellulose is negligible, which further demonstrates the advantages of cellulose as a green material (Figure S11). Notably, in the categories of terrestrial ecotoxicity and eutrophication, P‐CNCs‐Ti^4+^ reduces environmental impacts by over 70‐fold compared to commercial materials. This improvement primarily originated from the biomass‐derived cellulose feedstock and a simplified synthesis process, aligning with green chemistry principles. For peptide separation materials, production cost is as critical as environmental impact. The total cost of P‐CNCs synthesis and Ti^4^^+^ chelation was calculated, accounting for raw materials, consumables, and utilities (Table S4 and Table S5). The developed material achieves a 76% reduction in production cost compared to commercial SPE‐Ti‐IMAC materials, amounting to less than 1,500 CNY per kg at industrial scale (Figure 7i–m). This cost advantage arises from the low‐cost cellulose feedstock and a streamlined synthesis process requiring fewer purification steps. In summary, P‐CNCs and P‐CNCs‐Ti^4^^+^ materials offer exceptional peptide separation performance, ultra‐low environmental impacts, and a 76% cost reduction, establishing a sustainable and economical platform for glycopeptide/phosphopeptide enrichment.

## 3 Conclusion

In summary, a sustainable one‐step strategy is presented for the synthesis of P‐CNCs, enabling dual‐affinity enrichment for glycoproteomics and phosphoproteomics. This green and scalable approach simultaneously introduces phosphate functionalities and generates nanocrystals through phosphoric acid hydrolysis, eliminating complex multi‐step syntheses.

P‐CNCs exhibit outstanding analytical performance, enabling the identification of 2,025 N‐linked and 2,183 O‐GalNAc intact glycopeptides from only 1 µL of human serum, revealing novel glycan chemical modifications, as well as site‐specific fucosylation patterns on immunologically relevant proteins. For phosphoproteomics, P‐CNCs‐Ti^4+^ outperformed commercial TiO_2_‐based materials, enabeling identification of 5,225 phosphopeptides and discovery of seven types of non‐canonical phosphorylation events, including functionally important 3‐phosphoglyceryl modifications on key glycolytic enzymes. Beyond analytical performance, the sustainability impact of the proposed platform is substantial. Life cycle assessment (LCA) revealed significantly reduced environmental burdens across all evaluated impact categories, accompanied by a 76% reduction in production cost compared to commercial enrichment materials. Collectively, these results establish P‐CNCs as a versatile and sustainable enrichment platform that integrates high‐performance proteomic analysis with environmental responsibility. By demonstrating that green nanomaterials can simultaneously advance analytical depth and sustainability, this work opens new opportunities for large‐scale PTM profiling, biomarker discovery, and mechanistic investigations in complex biological systems.

## 4 Experimental Sections

### 4.1 Preparation of the P‐CNCs and P‐CNCs‐Ti^4+^

P‐CNCs were prepared from MCC through hydrolysis with phosphoric acid, MCC powder (2 g), 85% phosphoric acid (49.5 mL), and H_2_O (10.5 mL) were heated to 60(C in an oil bath with stirring at 500 rpm. The reaction proceeded for 5 h. The system was then cooled to room temperature and ultrasonicated for 15 min in an ice bath, followed by centrifugation at 10 000 rpm for 5 min at room temperature to remove residual acids. The centrifugation was repeated for three cycles. Once the solution reached a pH of approximately 6. Subsequently, the suspension was poured into a dialysis membrane with a molecular weight cut‐off of 8000 Da and dialyzed against 4 volumes of water for 4–5 days (water was changed every 12 h) for purification. Finally. the P‐CNCs were incubated in 200 mg mL^−1^ Ti(SO_4_)_2_ solution overnight. The resulting material was washed with water and 200 mM NaCl aqueous solution (containing 0.1% TFA) several times to remove dissociated Ti^4+^.

### 4.2 Characterization of the P‐CNCs and P‐CNCs‐Ti^4+^

Transmission electron microscopy (TEM) observations were performed using a JEM‐1400 Plus transmission electron microscope (JEOL, Japan). Samples were prepared by depositing 10 µL of a 0.01 wt.% P‐CNCs aqueous suspension onto glow‐discharged 300‐mesh copper grids coated with a Formvar/carbon film (Ted Pella, Inc., USA), followed by negative staining with 0.2 wt.% phosphotungstic acid prior to imaging.

X‐ray diffraction (XRD) patterns of P‐CNCs were collected on a Bruker D8 ADVANCE diffractometer equipped with a LynxEye detector, using Cu Kα radiation (λ = 1.5406 Å, 40 kV, 40 mA). Data were acquired in Bragg–Brentano geometry over a 2θ range of 10°– 40° with a step size of 0.02° and scan rate of 2°/min.

X‐ray photoelectron spectroscopy (XPS) analysis was conducted using a Thermo Scientific K‐Alpha+ spectrometer with a monochromatic Al Kα X‐ray source (1486.6 eV). Survey scans were acquired at 160 eV pass energy and high‐resolution regional scans at 40 eV pass energy, with charge neutralization applied and base pressure maintained below 5 × 10^−^^8^ mbar.

Zeta potential measurements were performed on P‐CNCs and P‐CNCs‐Ti^4+^ suspensions using a Malvern Zetasizer Nano ZS90 instrument (dynamic light scattering mode, 25°C).

### 4.3 Enrichment of N‐Glycopeptides With P‐CNCs

Prior to enrichment, P‐CNCs were washed three times with loading buffer (400 µL × 3, ACN/H_2_O/TFA, 78/21/1, v/v/v). Subsequently, the washed P‐CNCs were incubated with 30 µg IgG or 120 µg serum tryptic digests dissolved in loading buffer (400 µL, ACN/H_2_O/TFA, 78/21/1, v/v/v). The mixture was sonicated for 5 min to disperse the material as much as possible. A bioshaker was employed to incubate the mixture at 25°C for 30–60 min. Subsequently, the mixture was centrifuged at 15 000 *g* for 5 min to remove the solution, and a washing buffer (600 µL × 3, ACN/H_2_O/TFA, 78/21/1, v/v/v) was used to wash the residual substance for 5 min 3 times. The elution buffer (200 µL × 2, ACN/H_2_O/FA, 30/69/1, v/v/v) was used to elute the captured glycopeptides for 10 min at 25°C twice. Finally, MALDI‐TOF MS was employed to analyze the eluted solution with a simple sample. For complex serum samples, the eluates were collected, desalted, vacuum‐dried, and analyzed by liquid chromatography mass spectrometry (LC‐MS).

### 4.4 Enrichment of O‐GalNAc Glycopeptides With P‐CNCs

Prior to O‐glycopeptide enrichment, serum tryptic glycopeptides were treated with PNGase F to selectively remove N‐linked glycans. After N‐glycan removal, the resulting peptides were subjected to O‐GalNAc glycopeptide enrichment using P‐CNCs following the same procedure described in Section 4.2.

### 4.5 Enrichment of Phosphopeptides With P‐CNCs‐Ti^4+^

Prior to enrichment, P‐CNCs‐Ti^4+^ were washed three times with loading buffer (400 µL × 3, ACN/H_2_O/TFA, 80/14/6, v/v/v). Subsequently, the 16 µg β‐casein or 500 µg mouse liver digests were first redissolved in 200 µL of 100 mM NH4HCO3, and then the loading buffer (200 µL, ACN/H2O/TFA, 80/14/6, v/v/v) was added. The mixture was sonicated for 5 min to disperse the material as much as possible. A bioshaker was employed to incubate the mixture at 25°C for 30–60 min. Subsequently, the mixture was centrifuged at 15 000 g for 5 min to remove the solution, The remaining material was sequentially washed with washing buffer 1 (600 µL × 2, ACN/400 mM NaCl/TFA, 50/50/6, v/v/v) for 30 min each, followed by washing buffer 2 (600 µL, ACN/H_2_O/TFA, 30/79.9/0.1, v/v/v) for 30 min. The elution buffer (200 µL × 2, 10% NH_3_·H_2_O) was used to elute the captured phosphopeptides for 30 min at 25°C twice. Finally, MALDI‐TOF MS was employed to analyze the eluted solution with a simple sample. For complex biological samples, including mouse liver digests, the eluates were collected, desalted, and vacuum‐dried. And analyzed by LC‐MS.

### 4.6 LC‐MS/MS Analysis

The peptides were solubilized using 20 µL of 0.1% FA (v/v), and a portion of the peptide was injected into an LC–MS/MS system consisting of a Dionex UltiMate 3000 liquid chromatography and a Q Exactive HF mass spectrometer (Thermo Fisher Scientific, USA).

Peptide separation was performed on a capillary analytical column (15 cm length, 150 µm i.d.) packed in‐house with reverse phase C18 resins (1.8 µm particle size, 150‐Å pore size, Dr Maisch GmbH, Germany) with a 90 min gradient from 4% to 72% (v/v) ACN in 0.1% (v/v) FA. Spectra (AGC target 3 × 10^6^ and maximum injection time of 36 ms) were collected from 350 to 1 800 m/z at a resolution of 60 000 followed by data‐dependent HCD MS/MS (at a resolution of 15 000, stepped collision energy of 30 ± 5, intensity threshold of 5.8 × 10^3^, and maximum IT of 120 ms) of the 20 most abundant ions using an isolation window of 1.6 m/z.

### 4.7 Data Analysis

Intact N‐linked glycopeptides from human serum were identified and characterized using Glyco‐Decipher software (version 1.04) by searching against the human UniProt database (20,349 protein entries, release 2020_06) [41]. Trypsin was specified as the digestion enzyme with up to three missed cleavages allowed. Carbamidomethylation of cysteine (C, +57.022 Da) was set as a fixed modification, while oxidation of methionine (M, +15.995 Da) was set as a variable modification. The mass tolerances for precursor and fragment ions were set to 10 and 20 ppm, respectively. The false discovery rate (FDR) was controlled at 1% at both the peptide‐spectrum match (PSM) and peptide levels.

O‐GalNAc glycopeptides were identified using MS‐Decipher software by searching against the human UniProt database (20,349 protein entries, release 2020_06) [59]. Raw MS data were converted to MGF format prior to database searching. Database searches were performed using semi‐tryptic digestion, allowing up to three missed cleavages. The precursor and fragment ion mass tolerances were set to 10 ppm and 20 ppm, respectively. Precursor ion charge states ranging from +2 to +6 and peptide lengths of 6–46 amino acids were considered. Carbamidomethylation of cysteine (C, +57.022 Da) was specified as a fixed modification, while oxidation of methionine (M, +15.995 Da) and deamidation of asparagine and glutamine (N/Q, +0.984 Da) were specified as variable modifications. Peptide identifications were filtered using an FDR threshold of <1%.

Phosphopeptides were identified using pFind software (version 3.1.5) by searching against the mouse UniProt database (17,228 protein entries, release 2024_12) [60]. Trypsin was specified as the digestion enzyme with up to three missed cleavages allowed. Carbamidomethylation of cysteine (C, +57.022 Da) was set as a fixed modification, while oxidation of methionine (M, +15.995 Da) and phosphorylation of serine, threonine, and tyrosine (S/T/Y, +79.966331 Da) were set as variable modifications. To identify non‐canonical phosphorylated modifications, an open‐search strategy was employed by including the following variable modifications: Dimethylphosphothione (K, +123.974787 Da), 3‐phosphoglyceryl (K, +167.982375 Da), N‐dimethylphosphate (S, +107.013615 Da), Methylphosphonate (Y, +77.987066 Da), O‐Et‐N‐diMePhospho (S, +135.044900 Da), and PhosphoHex (S, +242.019154 Da). The mass tolerances for both precursor and fragment ions were set to 20 ppm, and the FDR threshold was controlled at 1%.

## Conflicts of Interest

The authors declare no conflict of interest.

## Supporting information




**Supporting File 1**: advs74615‐sup‐0001‐SuppMat.docx.

**Supporting File 2**: advs74615‐sup‐0002‐Data.zip.

## Data Availability

The data that support the findings of this study are available in the supplementary material of this article.
