# Supplementary material for: Sustainable Phosphorylated Cellulose Nanocrystals: A Dual‐Affinity Platform for High‐Efficiency Enrichment of Intact Glycopeptides and Phosphopeptides
Source: Adv Sci (Weinh). 2026 Feb 27;13(27):e23858. doi: 10.1002/advs.202523858 (PMC13170199; doi:10.1002/advs.202523858)
Supplement: Supplementary file 1 — Supporting File 1: advs74615‐sup‐0001‐SuppMat.docx. [file ADVS-13-e23858-s002.docx]

Supporting Information

**Sustainable Phosphorylated Cellulose Nanocrystals: A Dual-Affinity Platform for High-Efficiency Enrichment of Intact Glycopeptides and Phosphopeptides**

Jiaying Li^a, b, c^, Wuming Fan^a^, Xuyang Yue^c^, Dongdong Wang^a^, Xiangyu Tang^a^, Zhuo Zhang^b,d^, Yonggui Wang^a,^ *, Yanjun Xie^a,^ *, Mingliang Ye ^c,^ * and Hongqiang Qin^b, c,^ *

**Supplementary methods and data processing**

**Materials**

MCC (granule size = 25 μm) was purchased from guidechem (China), phosphoric acids, trifluoroacetic acid (TFA, 99%), formic acid (FA), dithiothreitol (DTT, 99%), iodoacetamide (IAA, 99%), 2,5-dihydroxybenzoic acid (DHB, 98%), β-casein, bovine serum albumin (BSA, 98%), Immunoglobulin G (IgG, 98%), trypsin, ammonium hydrogen carbonate (NH_4_HCO_3_), and urea were purchased from Sigma-Aldrich (St Louis, MO, USA). PNGase F was purchased from New England Biolabs (Ipswich, MA, USA). Acetonitrile (ACN, HPLC grade) and ammonium hydroxide (NH₃·H₂O, 25 wt% solution in water) were purchased from Merck (Darmstadt, Germany). The human serum and mouse liver for the experiment were obtained from Dalian University of Technology (Dalian, China). The samples were stored at -80 ℃ until usage. Click maltose-HILIC beads was prepared in house as reported.^[1]^

**pH Stability Evaluation by TEM**

To evaluate the pH stability of P-CNCs, the samples were dispersed in aqueous solutions with pH values of 2, 4, 10, and 12, respectively, and incubated for at least 8 h at room temperature. After incubation, aliquots of each suspension were diluted with deionized water to a final concentration of 0.01 wt% for TEM observation.

TEM imaging was performed using a JEM-1400 Plus transmission electron microscope (JEOL, Japan). Samples were prepared by depositing 10 μL of the diluted P-CNCs suspension onto glow-discharged 300-mesh copper grids coated with a Formvar/carbon film (Ted Pella, Inc., USA), followed by negative staining with 0.2 wt% phosphotungstic acid prior to imaging.

**Digestion of proteins**

β-Casein was solubilized in 100 mM NH_4_HCO_3_, and then maintained at 37 °C for 18 h with trypsin at an enzyme to protein ratio of 1:25 (wt/wt). Tryptic digests of β-casein were then divided into several parts and stored at −20 °C for further use. IgG was solubilized in 8 M urea/100 mM NH_4_HCO_3_, 10 mM DTT was added to the solution at 37 ℃ for 120 min or 56 ℃ for 60 min, and 20 mM iodoacetamide (IAA) was added in the dark for 30 min. Following alkylation, the proteins were diluted to a final 1 M urea with 100 mM NH_4_HCO_3_. Then, the proteins were digested with trypsin in a digestion buffer (100 mM NH_4_HCO_3_, pH 8.2) at 37 ℃ overnight. Trifluoroacetic acid (TFA) was added to quench the digestion reaction. The peptides were desalted using C18 Sep-Pak cartridge (Waters) and dried under vacuum for further enrichment.

The mouse liver was resuspended in 8 M urea and lysed by sonication. After centrifugation (1000 g, 5 min), the supernatant was collected and subjected to tryptic digestion following the above protocol used for IgG.

The serum was solubilized in 8 M urea/100 mM NH_4_HCO_3_, the supernatant was collected and subjected to tryptic digestion following the above protocol used for IgG. Peptides were desalted using C18 Sep-Pak cartridges (Waters) and vacuum-dried for N-Glycopeptides enrichment. After lyophilization, peptides were redissolved in Glycobuffer 2 (pH 7.5), followed by PNGase F treatment (3,000 U, 37°C/overnight) to release N-glycans. The digest was finally desalted with Oasis HLB SPE columns and lyophilized for O-GalNAc Glycopeptides enrichment.

**Enrichment of** **N-Glycopeptides with click maltose-HILIC**

The N-Glycopeptides were enriched by using the centrifugation assisted click maltose-HILIC approach according to our previous report.^[2]^ Firstly, 5 mg click maltose material were washed three times with (400 μL × 3, ACN/H_2_O/TFA, 80/19/1, v/v/v), and the digested proteins were re-dissolved in the loading buffer (400 μL, ACN/H_2_O/TFA, 80/19/1, v/v/v). The digests were mixed with the HILIC materials and the resulting suspension was shaken for 45 minutes. Then, the mixture was pipetted into a HILIC tip and washed with 40 μL of loading buffer twice. Finally, the enriched N-Glycopeptides were eluted with (100 μL × 2, ACN/H2O/FA, 30/69/1, v/v/v). After that the elution was combined and lyophilized for MS analysis.

**Matrix-Assisted Laser Desorption/ Ionization Time of Flight Mass Spectrometry (MALDI-TOF MS) analysis**

MALDI-TOF MS analysis was performed on a 5800 MALDI-TOF/TOF™ mass spectrometer (AB SCIEX) equipped with a 355 nm pulsed Nd:YAG laser. The matrix solution consisted of 25 mg mL⁻¹ 2,5-dihydroxybenzoic acid (DHB) dissolved in an ACN/H₂O/H₃PO₄ solvent mixture (70/29/1, v/v/v). An aliquot of 1 µL of the sample solution was spotted onto a MALDI target plate and air-dried at room temperature. Subsequently, 1 µL of the DHB matrix solution was applied onto the dried sample spot and allowed to air-dry before mass spectrometric analysis.

**Gene Ontology (GO) enrichment analysis**

GO enrichment analysis was performed in R using clusterProfiler.^[3]^ The input gene list consisted of proteins identified from O-glycoproteome with FDR < 1%. The background universe was defined as the human UniProt database (20,349 protein entries, release 2020_06).

**Sequence motif analysis**

For phosphopeptides identified from mouse liver and N-glycopeptides identified from human serum, amino acid sequences flanking the modification sites were extracted. The sequence motifs surrounding phosphorylation sites and N-glycosylation sites were analyzed using WebLogo to evaluate residue preferences and consensus patterns.^[4]^

**Simulation details and methods**

In the present work, the adsorption of three glycopeptides by phosphate modified cellulose crystals was studied using molecular dynamics simulations. Simulations were performed using the Gromacs-2023.5 software package and the molecular force field was performed using GAFF2.^[5-6]^ The results were visualized using the visualization software VMD. The detailed composition of each simulation system is summarized below. System 1 contained 10 P-CNCs, 5 oligomannose-type glycopeptides, 5 hybrid-type glycopeptides, and 5 complex-type glycopeptides. The solvent environment consisted of 8,814 acetonitrile (ACN) molecules and 2,373 water (H₂O) molecules, and 145 Na⁺ ions were included to maintain electroneutrality/ionic balance. All simulations were carried out with Gromacs 2023.5, using the GAFF2 force field, and the trajectories were visualized with VMD.

The non-bonding interactions contain van der Waals and electrostatic interactions, both of which have a cut-off radius set to r_c_ =1.2 nm. The interaction between particles is expressed using the LJ potential.

$$V_{\mathrm{LJ}}=\left\{ \begin{aligned} 4\epsilon[{(\frac{\sigma}{r})}^{12}-{(\frac{\sigma}{r})}^{6}], r<r_{c} \\ 0, r\geq r_{c} \end{aligned} \right.$$

Where $\epsilon$ and $\sigma$ denote the strength of the inter-particle interaction and the particle size, respectively, depending on the force field chosen. The electrostatic interaction between particles is expressed as the Coulomb potential:

$$V_{\mathrm{Coulomb}}=\left\{ \begin{aligned} \frac{1}{4\pi\varepsilon_{0}}\frac{q_{i}q_{j}}{\varepsilon_{r}r}, r<r_{c} \\ 0, r\geq r_{c} \end{aligned} \right.$$

Where $\varepsilon_{0}$ and $\varepsilon_{r}$ denote the vacuum dielectric constant and relative dielectric constant, and q denotes the charge carried by the particle. Restrained Electrostatic Potential (RESP) method is used to calculate atomic charges.^[7]^ Long range electrostatic interactions are treated using the particle mesh Ewald (PME) method.^[8]^

First, energy minimization was performed using the steepest descent method with energies and forces of 0.01 kJ/mol and 1000 kJ/mol/nm. Next, MD was run for 125,000 steps of 1fs, which is a constant volume process using a Berendsen thermostat. MD integrates Newton's equations using the velocity-Verlet algorithm. Then MD is run for 250,000 steps of 2 fs, using Berendsen for both the thermostat and pressure coupling. Finally, MD is run for 50,000,000 steps of 2 fs, using V-rescale and C-rescale for the thermostat and pressure coupling, respectively. The time constants for temperature coupling and pressure coupling are 1 ps and 2 ps, respectively. Simulations use periodic boundary conditions (PBC). The temperature of the simulated system is 298.15 K and the pressure is 1 atm.

**Life Cycle Assessment**

The environmental impact assessment within this Life Cycle Assessment (LCA) study was conducted using the CML-IA baseline methodology, version 3.08, developed by the Institute of Environmental Sciences (CML) at Leiden University. Based on the CML-IA methodology, this study is conducted following the steps outlined in ISO 14040 and ISO 14044.^[9]^ This scientific midpoint approach quantifies potential environmental impacts across multiple categories using characterization factors derived from underlying cause-effect pathways. The LCA data for upstream processes (chemicals, water, energy) were primarily sourced from the Ecoinvent database. The following impact categories were assessed according to the CML-IA baseline V3.08 guidelines: Abiotic Depletion (elements), Abiotic Depletion (fossil fuels), Global Warming Potential (GWP-100 years), Ozone Layer Depletion, Human Toxicity, Freshwater Aquatic Ecotoxicity, Marine Aquatic Ecotoxicity, Terrestrial Ecotoxicity, Photochemical Oxidation (Smog Formation), Acidification Potential, Eutrophication Potential. Characterization factors specific to each impact category were applied to the life cycle inventory (LCI) data to calculate the respective category indicator results. The functional unit for this comparative study is the production of 1 kg of SPE-Ti-IMAC and P-CNCs-Ti^4+^.


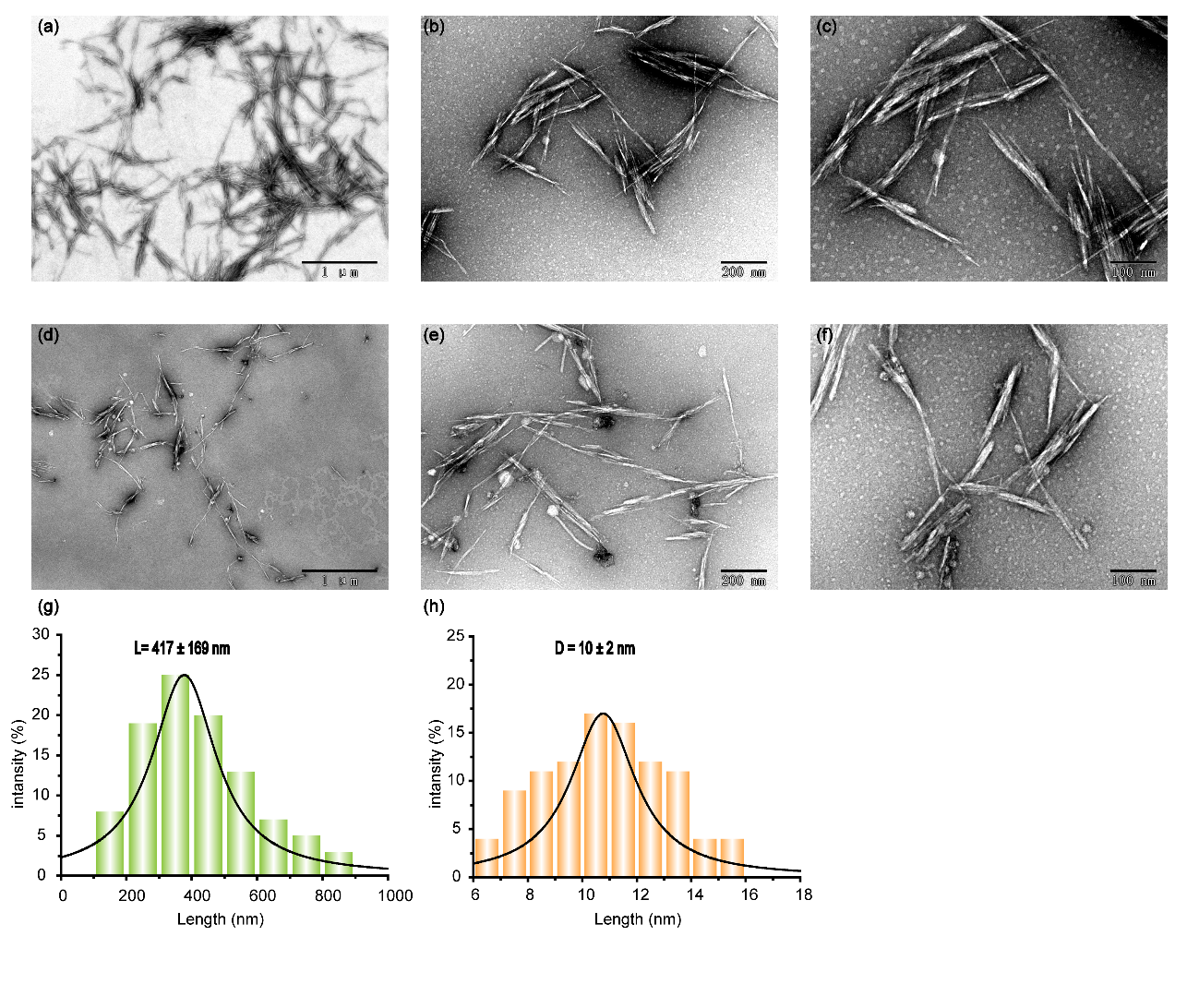


**Figure S1**. Transmission electron microscopy (TEM) images of P-CNCs (a-c) and P-CNCs–Ti⁴⁺ (d-f). Size distribution histograms of P-CNCs-Ti^4+^ showing length (g) and diameter (h).


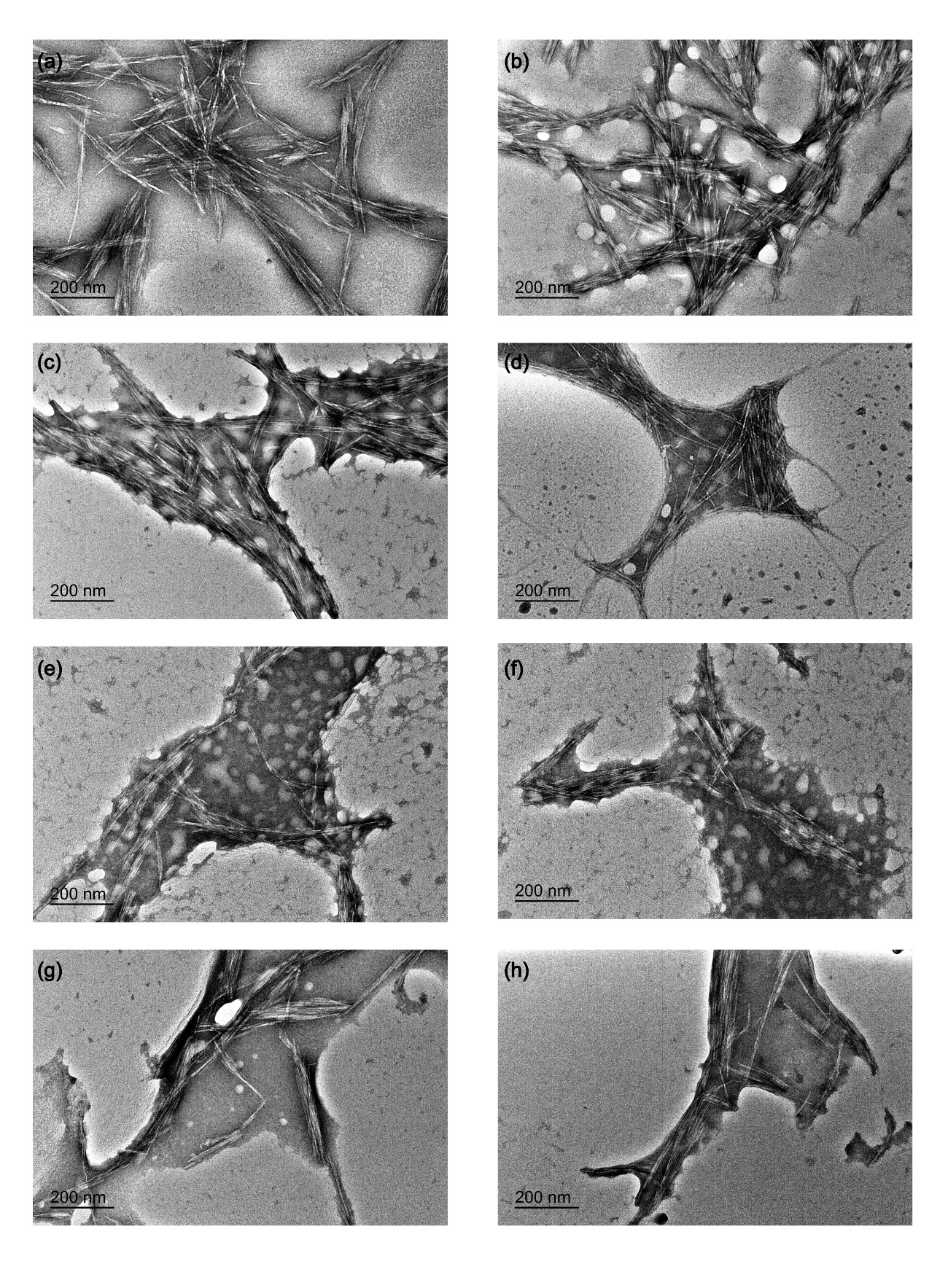


**Figure S2**. Transmission electron microscopy (TEM) images of P-CNCs after dispersion in aqueous solutions at different pH conditions: pH 2 (a-b), pH 4 (c-d), pH 10 (e-f), and pH 12 (g-h).


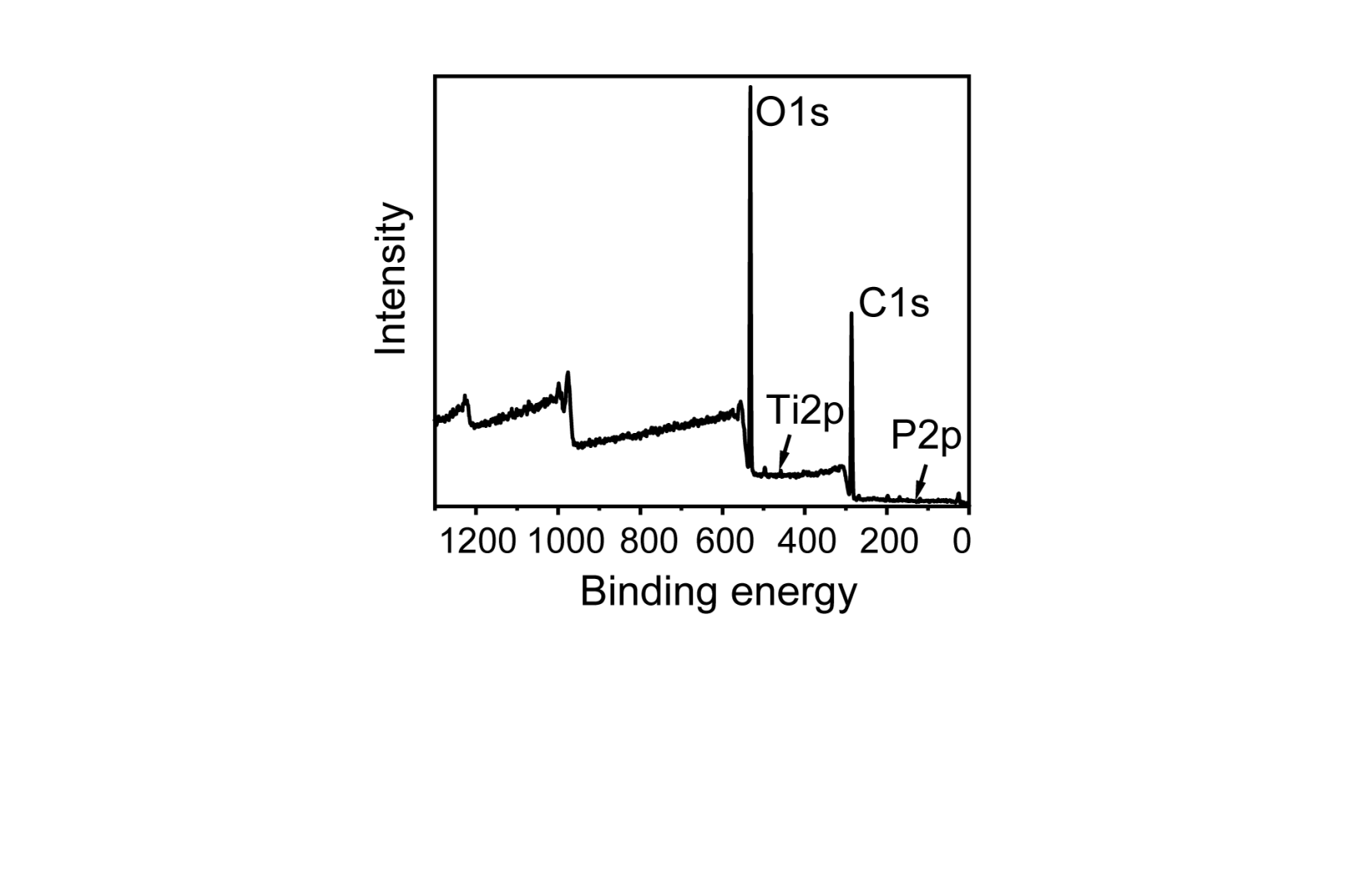


**Figure S3**. XPS full spectra of the P-CNCs-Ti^4+^.


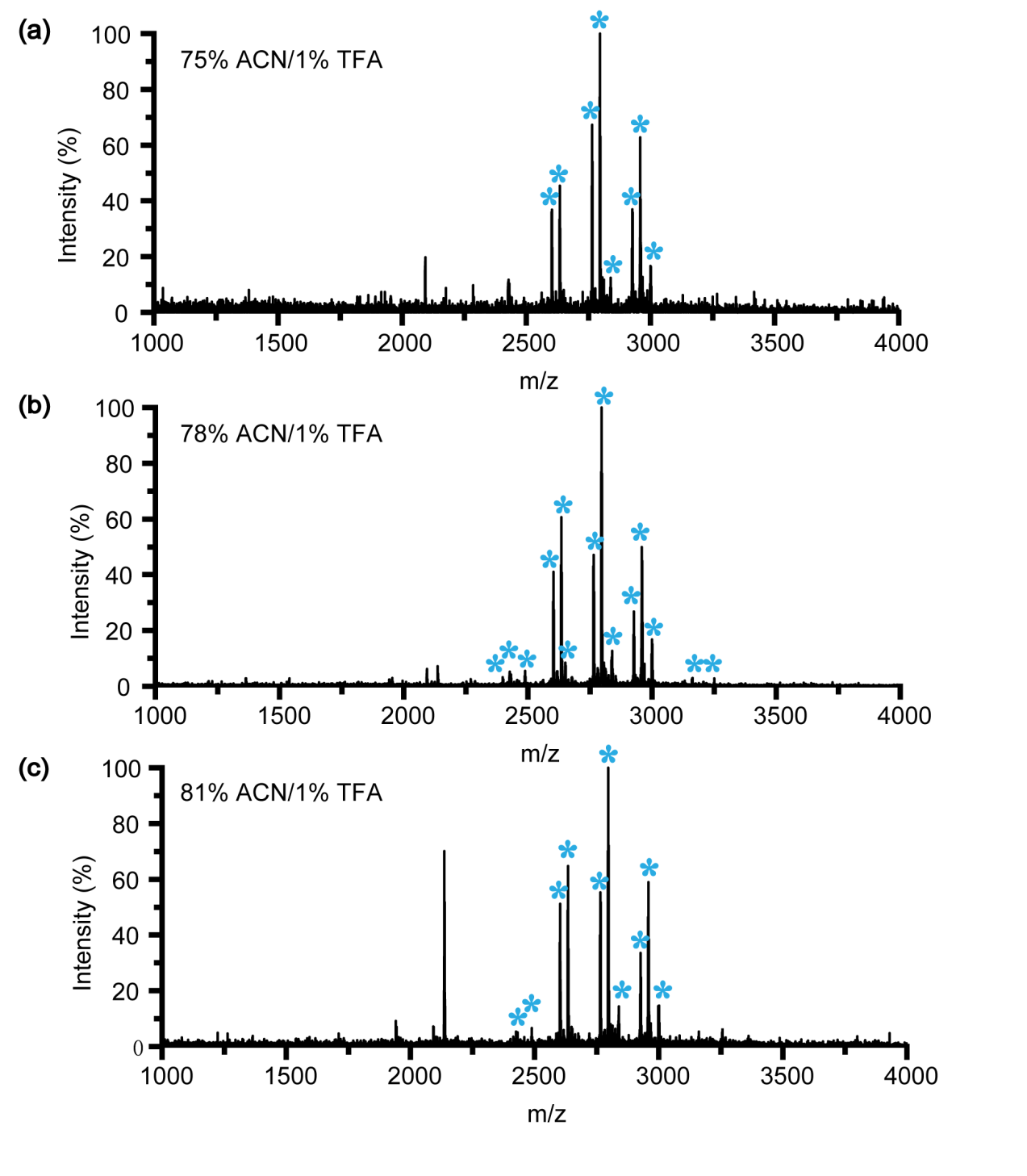


**Figure S4**. Matrix-Assisted Laser Desorption/ Ionization Time of Flight Mass Spectrometry (MALDI-TOF MS) spectra of elution fractions obtained using P-CNCs with different loading solutions. (a) acetonitrile (ACN) /H2O/trifluoroacetic acid (TFA) (75:24:1, v/v/v); (b) ACN/H2O/TFA (78:21:1, v/v/v); (b) ACN/H2O/TFA (81:18:1, v/v/v).


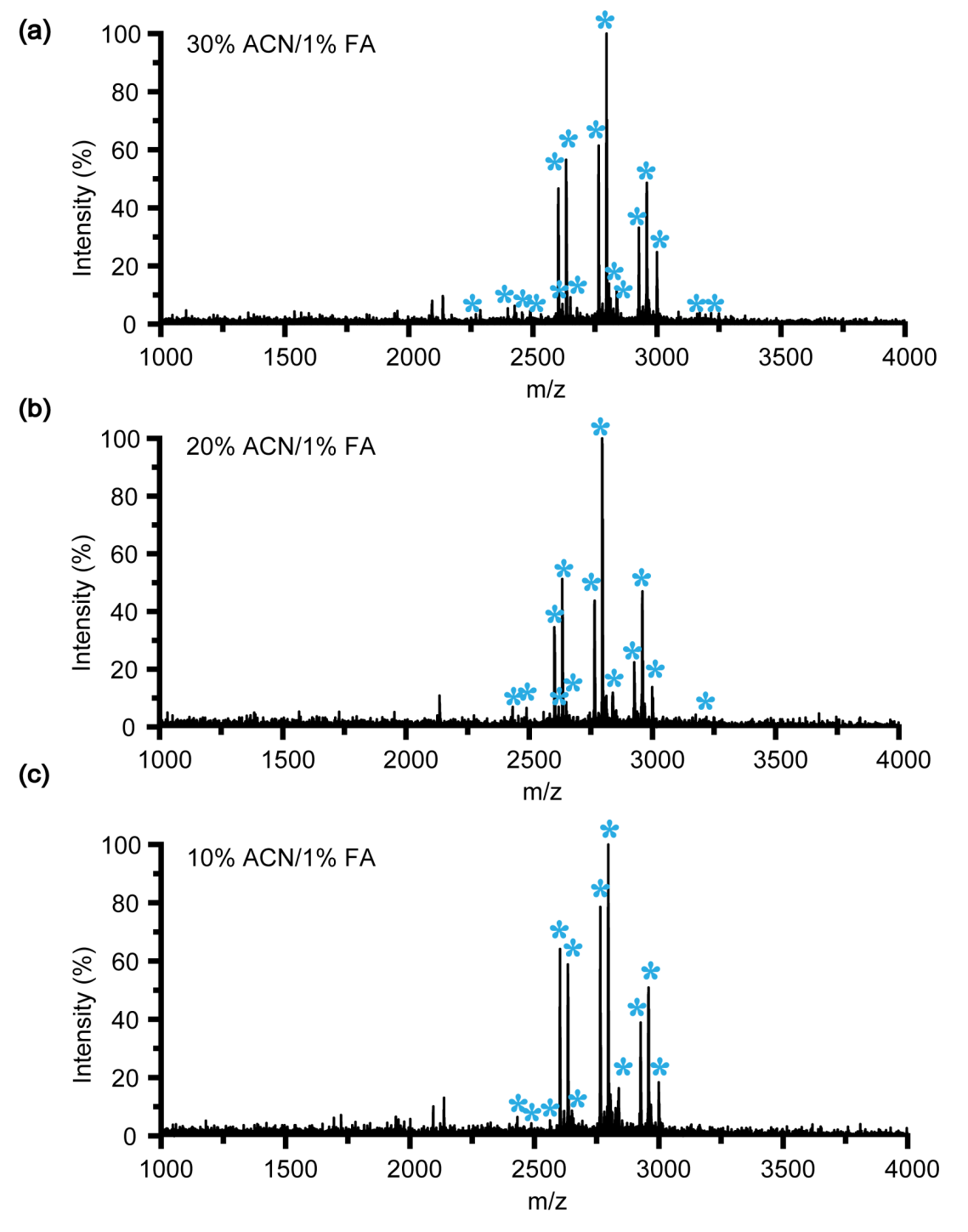


**Figure S5**. MALDI-TOF MS spectra of elution fractions obtained using P-CNCs with different elution solutions. (a) ACN/H2O/formic acid (FA)(30:69:1, v/v/v); (b) ACN/H2O/FA (20:79:1, v/v/v); (b) ACN/H2O/FA (10:89:1, v/v/v).


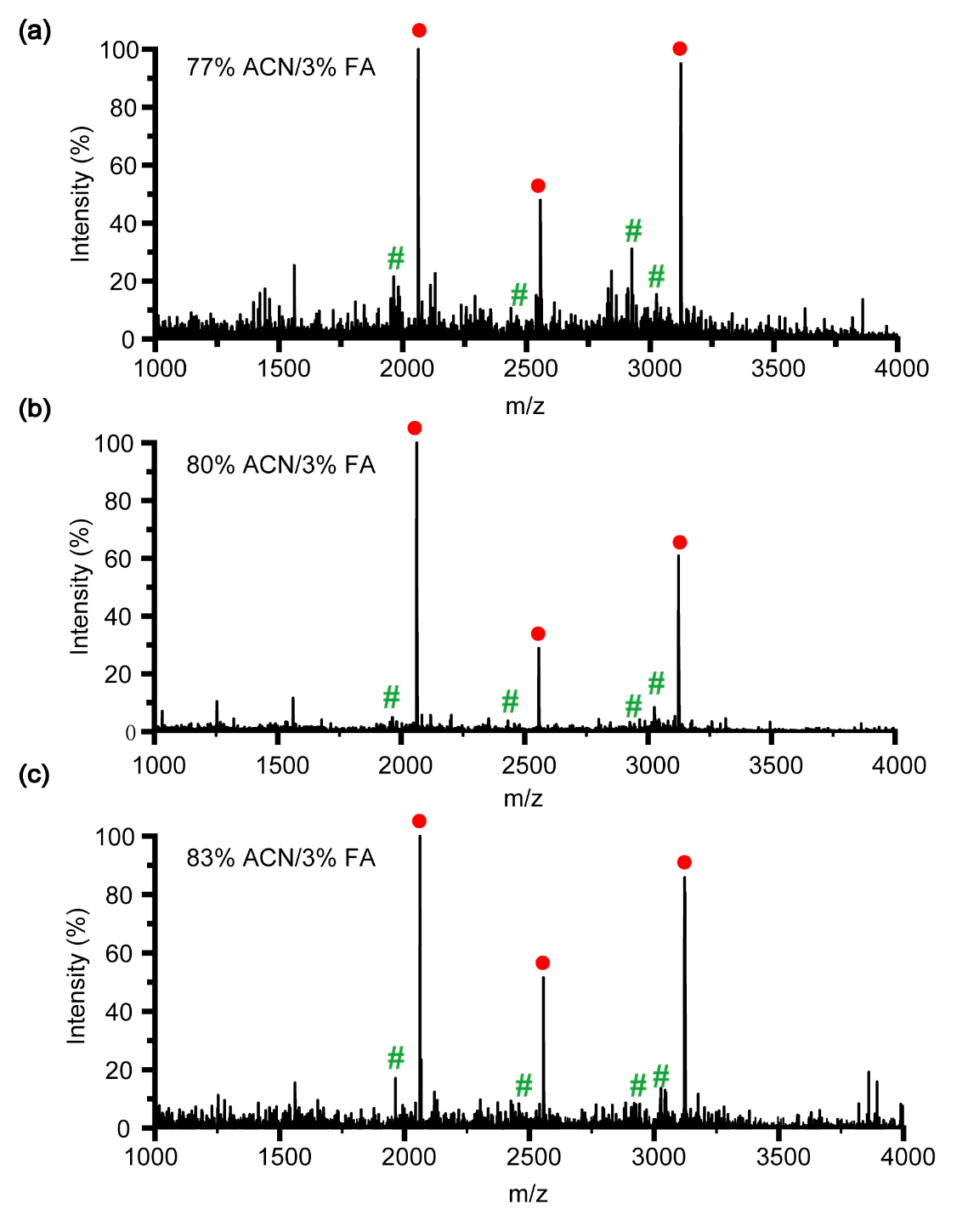


**Figure S6**. MALDI-TOF MS spectra of elution fractions obtained using P-CNCs-Ti^4+^ with loading solutions. (a) ACN/H2O/FA (77:20:3, v/v/v); (b) ACN/H2O/FA (80:17:3, v/v/v); (b) ACN/H2O/FA (83:14:3, v/v/v).


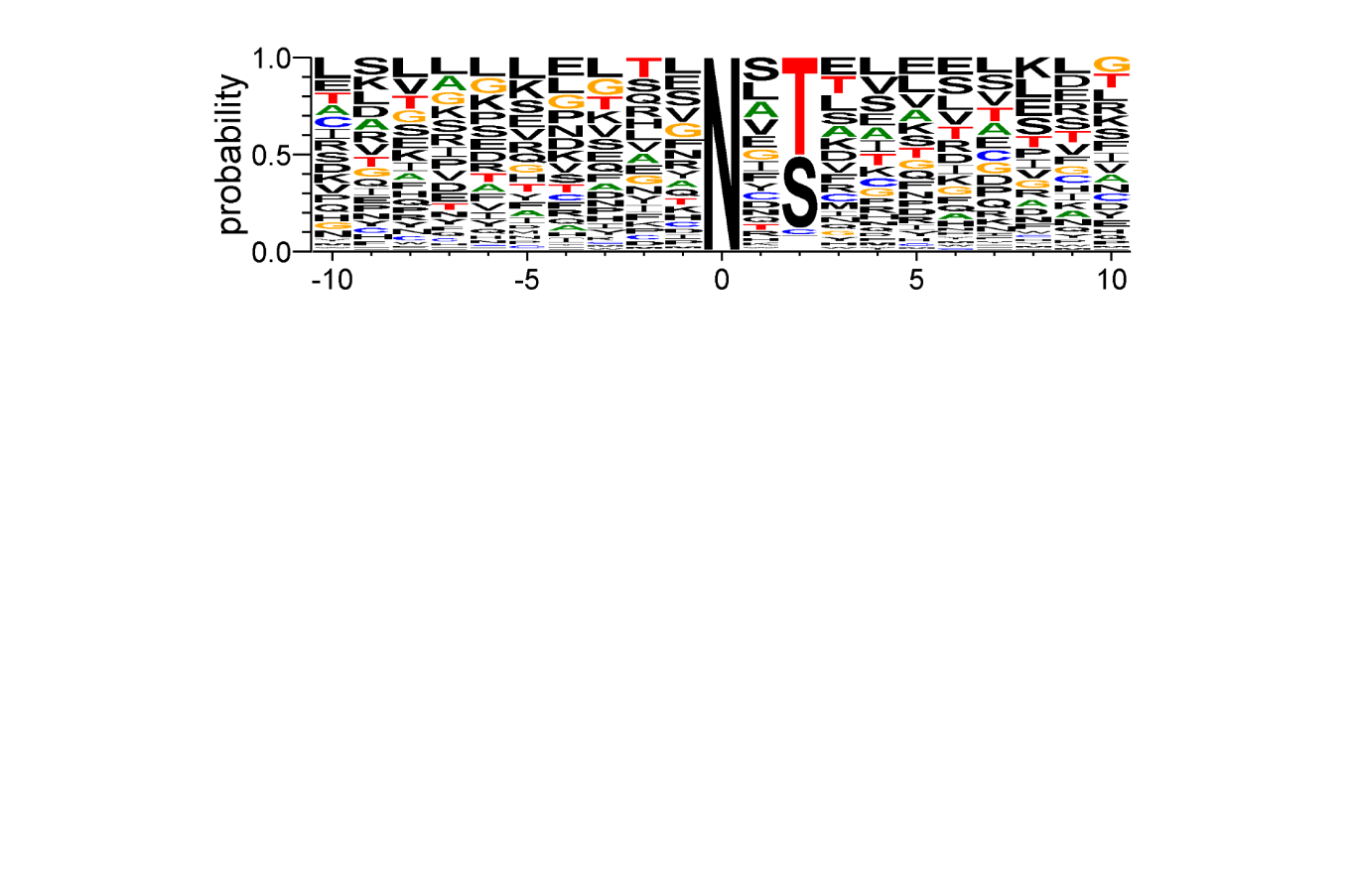


**Figure S7**. Motif analysis of identified N-linked glycopeptides.


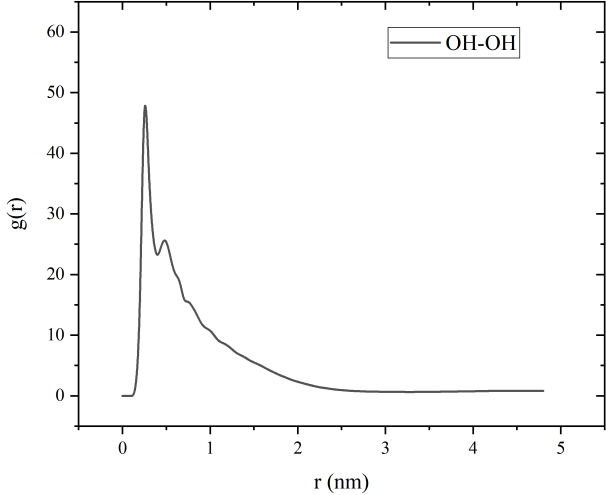


**Figure S8**. The radial distribution function (RDF) between OH groups at different locations on the P-CNCs was calculated.


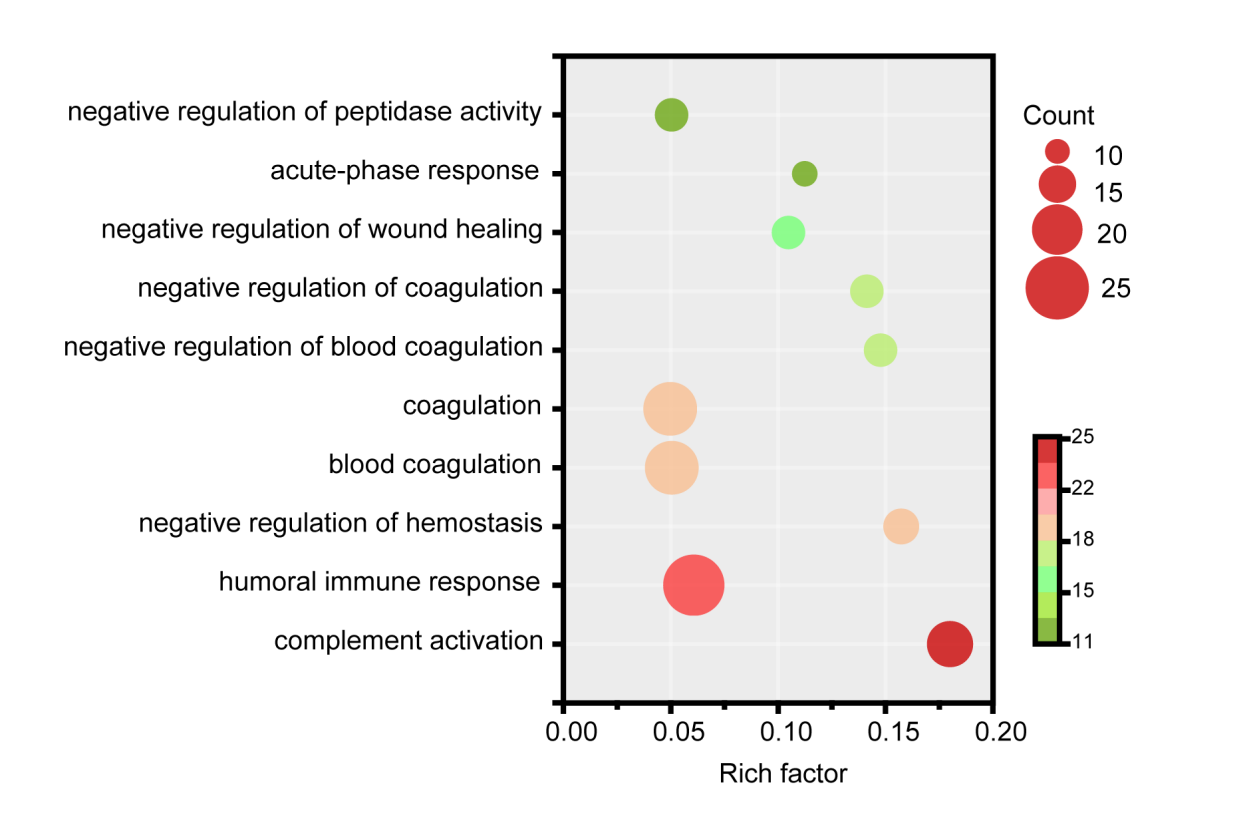


**Figure S9**. The gene ontology (GO) analysis of identified glycoproteins based on P-CNCs.


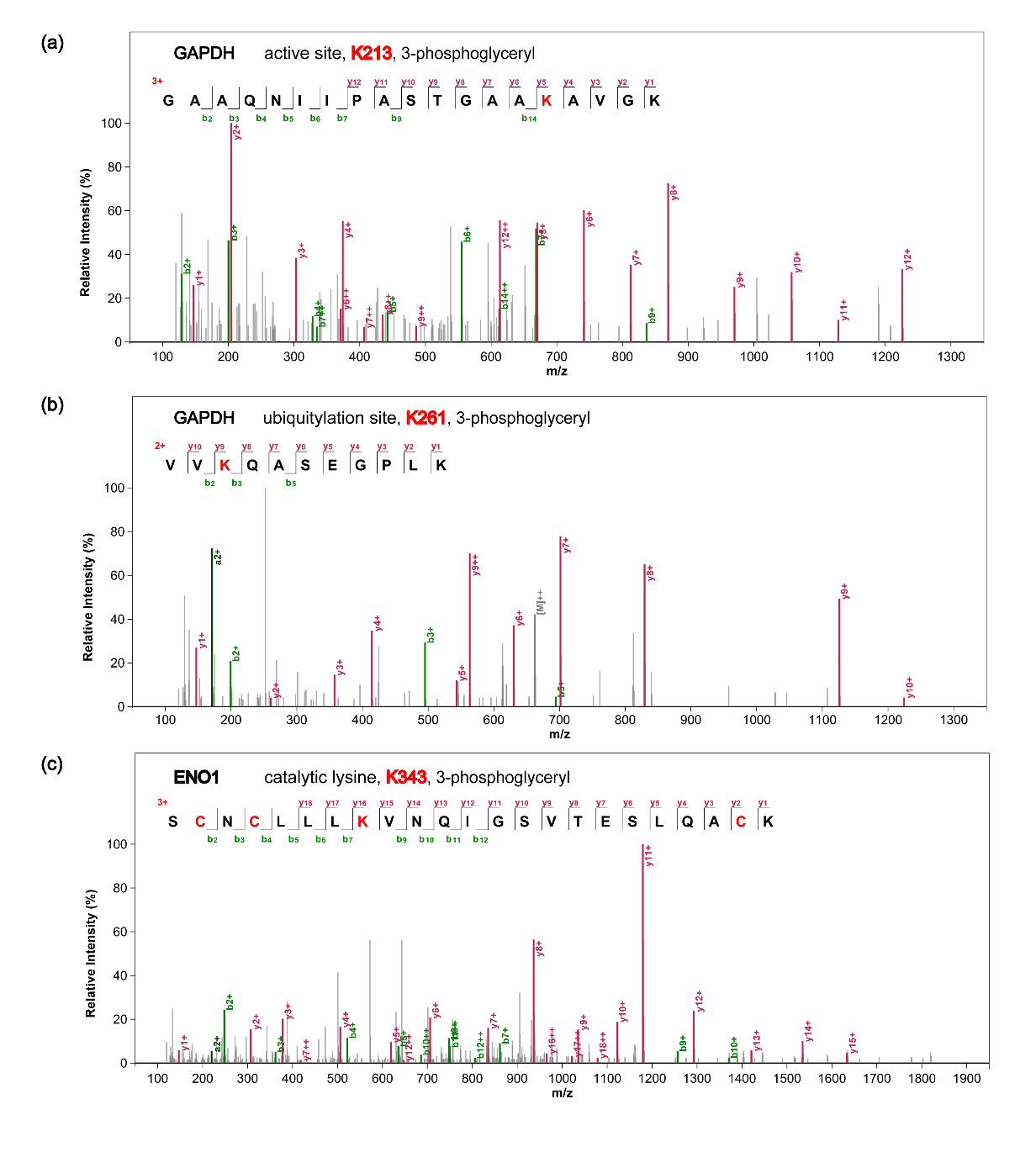


**Figure S10**. MS/MS spectra of 3-phosphoglyceryl [K] modified peptides. (a) Catalytic active site K213 and (b) ubiquitylation site K261 of glyceraldehyde-3-phosphate dehydrogenase (GAPDH); (c) catalytic lysine residue K343 of α-enolase (ENO1).


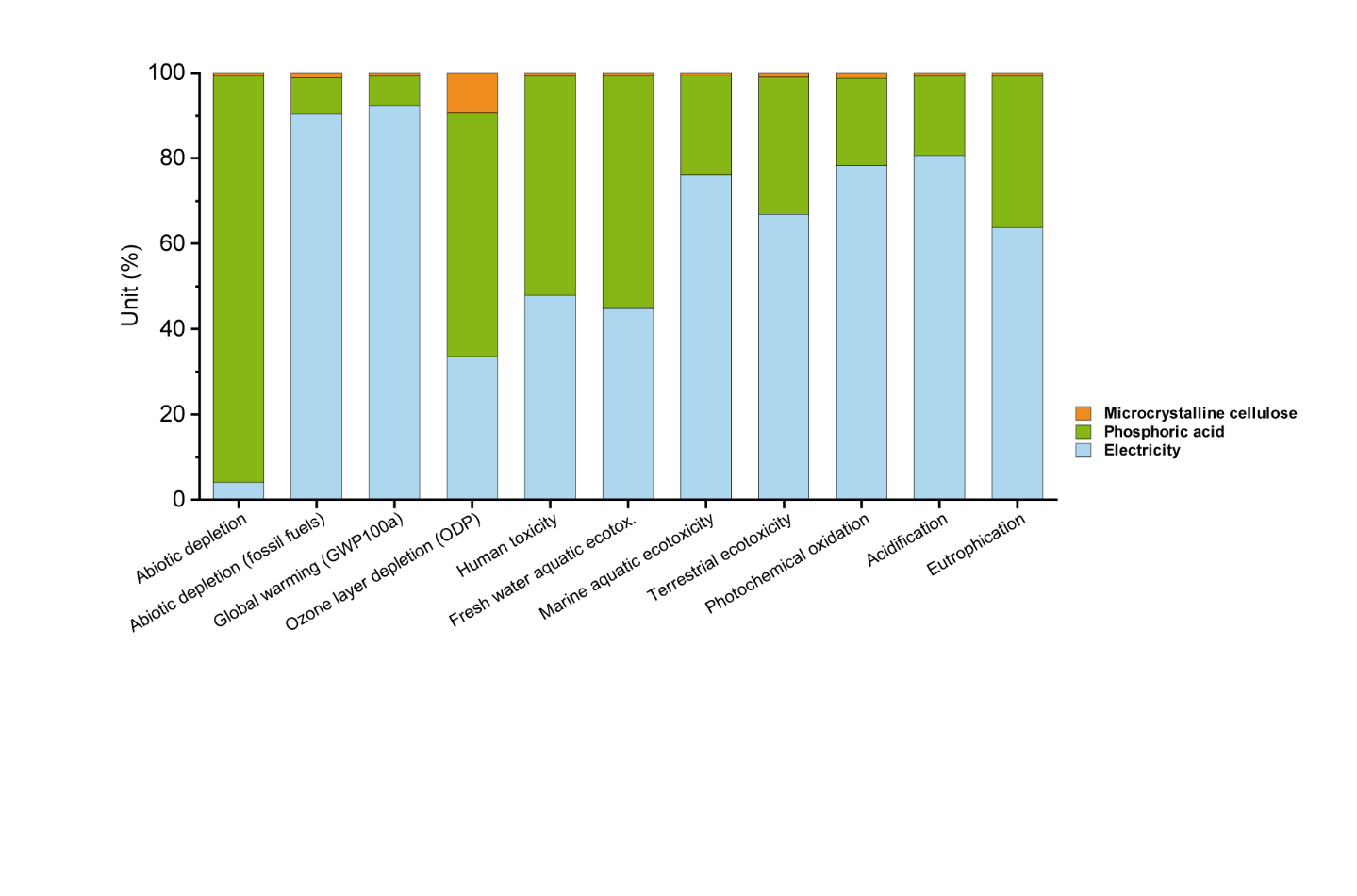


**Figure S11**. Contribution analysis results per kg of P-CNCs.

**Table S1.** Identified IgG glycopeptides detected using P-CNCs.

| **Number** | **Theoretical**  **glycopeptide m/z** | **Glycan composition** | **Peptide Sequence** |
| --- | --- | --- | --- |
| 1 | 2236.924 | (Hex)2 (NAc)3 (Fuc)1 | EEQFNSTFR |
| 2 | 2268.914 | (Hex)2 (NAc)3 (Fuc)1 | EEQYNSTYR |
| 3 | 2398.977 | (Hex)3 (NAc)3 (Fuc)1 | EEQFNSTFR |
| 4 | 2430.967 | (Hex)3 (NAc)3 (Fuc)1 | EEQYNSTYR |
| 5 | 2487.988 | (Hex)3 (NAc)4 | EEQYNSTYR |
| 6 | 2602.056 | (Hex)3 (NAc)4 (Fuc)1 | EEQFNSTFR |
| 7 | 2618.051 | (Hex)4 (NAc)4 | EEQFNSTFR |
| 8 | 2634.046 | (Hex)3 (NAc)4 (Fuc)1 | EEQYNSTYR |
| 9 | 2650.041 | (Hex)4 (NAc)4 | EEQYNSTYR |
| 10 | 2764.109 | (Hex)4 (NAc)4 (Fuc)1 | EEQFNSTFR |
| 11 | 2796.099 | (Hex)4 (NAc)4 (Fuc)1 | EEQYNSTYR |
| 12 | 2805.135 | (Hex)3 (NAc)5 (Fuc)1 | EEQFNSTFR |
| 13 | 2837.125 | (Hex)3 (NAc)5 (Fuc)1 | EEQYNSTYR |
| 14 | 2926.162 | (Hex)5 (NAc)4 (Fuc)1 | EEQFNSTFR |
| 15 | 2958.152 | (Hex)5 (NAc)4 (Fuc)1 | EEQYNSTYR |
| 16 | 2967.188 | (Hex)4 (NAc)5 (Fuc)1 | EEQFNSTFR |
| 17 | 2999.178 | (Hex)4 (NAc)5 (Fuc)1 | EEQYNSTYR |
| 18 | 3129.241 | (Hex)5 (NAc)5 (Fuc)1 | EEQFNSTFR |
| 19 | 3161.231 | (Hex)5 (NAc)5 (Fuc)1 | EEQYNSTYR |
| 20 | 3249.247 | (Hex)5 (NAc)4 (Fuc)1 (Sia)1 | EEQYNSTYR |

**Table S2. Identified β-casein phosphopeptides detected using P-CNCs-Ti^4+^.**

| **Number** | **Theoretical phosphopeptide m/z** | **Peptide Sequence** |
| --- | --- | --- |
| 1 | 2061.83 | FQ[pS]EEQQQTEDELQDK |
| 2 | 2556.09 | FQ[pS]EEQQQTEDELQDKIHPF |
| 3 | 3122.27 | RELEELNVPGEIVE[pS]L[pS][[pS][pS]EESITR |

**Table S3. Comparison of the enrichment performances of different materials for phosphopeptides from mouse liver**

| **Materials** | **Starting**  **amount** | **Identified**  **phosphopetides** | **Identified**  **phosphoproteins** | **Bifunctional** |
| --- | --- | --- | --- | --- |
| **Ti-PA-MNPs^[10]^** | 500ug | 2145 | 1271 | No |
| **commercial TiO_2_ kit^[11]^** | 500ug | 1568 | 925 | No |
| **TA-Ti-PA@Fe_3_O_4_^[12]^** | 500ug | 3408 | 1802 | No |
| **CS@PGMA@IDA^[13]^** | 50ug | 422 | 256 | Yes |
| **MagOTfP5SOF-Ga^3+[14]^** |  | 1332 | 908 | Yes |
| **This work** | 500ug | 5225 | 2343 | Yes |

**Table S4.** Raw material prices for P-CNCs production, sourced from commercial procurement websites.

| **Chemical** | **Purchase link** | **Quantity** | **Unit** | **Unit price (CNY)** | **Cost (CNY)** |
| --- | --- | --- | --- | --- | --- |
| **Microcrystalline cellulose** | https://china.guidechem.com/trade/pdetail29256455.html | 0.002 | kg | 10 | 0.02 |
| **Water** | Industrial water prices in China | 4.6955 | L | 0.00483 | 0.03 |
| **Phosphoric acids** | https://china.guidechem.com/cas/15369.html | 0.0495 | L | 11.8 | 0.58 |
| **Energy** | Electricity prices for industry in China | 1.1616 | kwh | 0.65 | 0.76 |
| **Dialysis membrane** | https://www.bjbiotopped.com/product/PNOMD4408.html | 16.5 | cm | 0.45 | 7.43 |
| **Titanium sulfate** | https://china.guidechem.com/cas/17722.html | 0.05 | kg | 65 | 3.25 |
| **Trifluoroacetic acid** | https://www2.caigoubang.com.cn/productlist.aspx?cbn=CB5127175 | 0.00126 | L | 1007 | 1.27 |
| **Sodium chloride** | https://china.guidechem.com/cas/15351.html | 0.004032 | kg | 7 | 0.03 |

**Table S5.** Raw material prices for SPE-Ti-IMAC production, sourced from commercial procurement websites.

| **Chemical** | **Purchase link** | **Quantity** | **Unit** | **Unit price (CNY)** | **Cost (CNY)** |
| --- | --- | --- | --- | --- | --- |
| **Polyvinyl Alcoho (PVA)** | https://www.chemicalbook.com/priceindex_cb7264573.htm | 0.001 | kg | 13.1 | 0.0131 |
| **Ethylene Glycol Dimethacrylate (EDMA)** | https://www.shyuanye.com/ | 0.015 | L | 728 | 10.92 |
| **Glycidyl Methacrylate (GMA)** | https://www.shyuanye.com/ | 0.02 | L | 0.22554 | 0.0045108 |
| **Benzoyl Peroxide (BPO)** | https://www.shyuanye.com/ | 0.00012 | kg | 180.8 | 0.021696 |
| **Toluene** | https://www.sigmaaldrich.cn/CN/zh/product/sial/244511 | 0.01 | L | 169.0522778 | 1.690522778 |
| **Nitrogen gas** | https://www.sci99.com/monitor-57237214-0.html | 0.25 | L | 0.964871194 | 0.241217799 |
| **Ethanol** | https://www.shyuanye.com/goods-W14395.html | 0.9 | L | 33.6 | 30.24 |
| **Ethylenediamine** | https://chem.100ppi.com/price/plist-1332-1.html | 0.15 | L | 9.802158273 | 1.470323741 |
| **Hydrochloric acid** | https://www.100ppi.com/mprice/plist-1-819-1.html | 0.01 | L | 0.733591138 | 0.007335911 |
| **Formaldehyde** | https://china.guidechem.com/cas/3.html | 0.008 | L | 138.4 | 1.1072 |
| **Methanol** | https://www.baiinfo.com/jiachunchanye/jiachun | 0.45 | L | 1.828795621 | 0.822958029 |
| **Titanium sulfate** | https://china.guidechem.com/cas/17722.html | 0.05 | kg | 65 | 3.25 |
| **Trifluoroacetic acid** | https://www2.caigoubang.com.cn/productlist.aspx?cbn=CB5127175 | 0.00126 | L | 1007 | 1.26882 |
| **Sodium chloride** | https://china.guidechem.com/cas/15351.html | 0.004032 | kg | 7 | 0.028224 |
| **Energy** | Electricity prices for industry in China | 6.79116 | kwh | 0.65 | 4.414254 |
| **Water** | Industrial water prices in China | 1.875 | L | 0.00483 | 0.00905625 |

**References**

[1] L. Yu, X. Li, Z. Guo, et al., "Hydrophilic Interaction Chromatography Based Enrichment of Glycopeptides by Using Click Maltose: A Matrix with High Selectivity and Glycosylation Heterogeneity Coverage," *Chemistry-a European Journal.* **2009**, *15* (46), 12618-12626.

[2] Y. Chen, H. Qin, X. Yue, et al., "Highly Efficient Enrichment of O-GlcNAc Glycopeptides Based on Chemical Oxidation and Reversible Hydrazide Chemistry," *Analytical Chemistry.* **2021**, *93* (49), 16618-16627.

[3] G. Yu, L.-G. Wang, Y. Han, et al., "clusterProfiler: an R Package for Comparing Biological Themes Among Gene Clusters," *Omics-a Journal of Integrative Biology.* **2012**, *16* (5), 284-287.

[4] G. E. Crooks, G. Hon, J. M. Chandonia, et al., "WebLogo: A sequence logo generator," *Genome Research.* **2004**, *14* (6), 1188-1190.

[5] H. J. C. Berendsen, D. Vanderspoel, R. Vandrunen, "GROMACS: A message-passing parallel molecular dynamics implementation," *Computer Physics Communications.* **1995**, *91* (1-3), 43-56.

[6] J. M. Wang, R. M. Wolf, J. W. Caldwell, et al., "Development and testing of a general amber force field," *Journal of Computational Chemistry.* **2004**, *25* (9), 1157-1174.

[7] J. M. Wang, P. Cieplak, P. A. Kollman, "How well does a restrained electrostatic potential (RESP) model perform in calculating conformational energies of organic and biological molecules?," *Journal of Computational Chemistry.* **2000**, *21* (12), 1049-1074.

[8] U. Essmann, L. Perera, M. L. Berkowitz, et al., "A smooth particle mesh Ewald method," *Journal of Chemical Physics.* **1995**, *103* (19), 8577-8593.

[9] M. Finkbeiner, A. Inaba, R. B. H. Tan, et al., "The new international standards for life cycle assessment:: ISO 14040 and ISO 14044," *International Journal of Life Cycle Assessment.* **2006**, *11* (2), 80-85.

[10] K. Zhang, D. Hu, S. Deng, et al., "Phytic acid functionalized Fe_3_O_4_ nanoparticles loaded with Ti(IV) ions for phosphopeptide enrichment in mass spectrometric analysis," *Microchimica Acta.* **2019**, *186* (2), 68.

[11] L. Zhang, Q. Zhao, Z. Liang, et al., "Synthesis of adenosine functionalized metal immobilized magnetic nanoparticles for highly selective and sensitive enrichment of phosphopeptides," *Chemical Communications.* **2012**, *48* (50), 6274-6276.

[12] K. N. Zhang, Y. Hao, D. H. Hu, et al., "Development of dual-ligand titanium (IV) hydrophilic network sorbent for highly selective enrichment of phosphopeptides," *Journal of Chromatography A.* **2021**, *1659*, 462648.

[13] X. J. Zou, J. Z. Jie, B. Yang, "Single-Step Enrichment of N-Glycopeptides and Phosphopeptides with Novel Multifunctional Ti^4+^-Immobilized Dendritic Polyglycerol Coated Chitosan Nanomaterials," *Analytical Chemistry.* **2017**, *89* (14), 7520-7526.

[14] H. J. Zheng, J. X. Jia, Z. Li, et al., "Bifunctional Magnetic Supramolecular-Organic Framework: A Nanoprobe for Simultaneous Enrichment of Glycosylated and Phosphorylated Peptides," *Analytical Chemistry.* **2020**, *92* (3), 2680-2689.
